# Numerical Responses of Saproxylic Beetles to Rapid Increases in Dead Wood Availability following Geometrid Moth Outbreaks in Sub-Arctic Mountain Birch Forest

**DOI:** 10.1371/journal.pone.0099624

**Published:** 2014-06-09

**Authors:** Ole Petter Laksforsmo Vindstad, Sabrina Schultze, Jane Uhd Jepsen, Martin Biuw, Lauri Kapari, Anne Sverdrup-Thygeson, Rolf Anker Ims

**Affiliations:** 1 Department of Arctic and Marine Biology, University of Tromsø, Tromsø, Norway; 2 Department of Ecology, Leuphana University Lüneburg, Lüneburg, Germany; 3 Norwegian Institute for Nature Research, Fram Centre, Tromsø, Norway; 4 Norwegian University of Life Sciences, Department of Ecology and Natural Resource Management, Aas, Norway; University of Waikato (National Institute of Water and Atmospheric Research), New Zealand

## Abstract

Saproxylic insects play an important part in decomposing dead wood in healthy forest ecosystems, but little is known about their role in the aftermath of large-scale forest mortality caused by pest insect outbreaks. We used window traps to study short-term changes in the abundance and community structure of saproxylic beetles following extensive mortality of mountain birch in sub-arctic northern Norway caused by an outbreak of geometrid moths. Three to five years after the outbreak, the proportion of obligate saproxylic individuals in the beetle community was roughly 10% higher in forest damaged by the outbreak than in undamaged forest. This was mainly due to two early-successional saproxylic beetle species. Facultative saproxylic beetles showed no consistent differences between damaged and undamaged forest. These findings would suggest a weak numerical response of the saproxylic beetle community to the dead wood left by the outbreak. We suggest that species-specific preferences for certain wood decay stages may limit the number of saproxylic species that respond numerically to an outbreak at a particular time, and that increases in responding species may be constrained by limitations to the amount of dead wood that can be exploited within a given timeframe (i.e. satiation effects). Low diversity of beetle species or slow development of larvae in our cold sub-arctic study region may also limit numerical responses. Our study suggests that saproxylic beetles, owing to weak numerical responses, may so far have played a minor role in decomposing the vast quantities of dead wood left by the moth outbreak.

## Introduction

Insect outbreaks are major disturbance factors in many forest ecosystems, periodically causing defoliation and mortality of trees and other vegetation across vast areas [1]. The potential causes of outbreaks have been intensively studied for decades. More recently, however, there has also been increasing interest in their ecological consequences [2]. One important consequence of outbreaks is that they alter resource availability for many consumer species. For instance, outbreaks can increase food availability for insectivorous birds [3]–[5], small mammals [6], [7] and invertebrates [8], [9]. Similarly, understory vegetation may benefit from increased nutrient inputs in the form of frass and insect cadavers [10], and increased availability of light due to removal of foliage in the canopy [11], [12]. The resulting increase in understory plant biomass may, in turn, provide resources for species that depend on understory vegetation for food or cover [13], [14]. In all of these cases, outbreaks provide consumers with resource pulses, i.e. rare and ephemeral events of resource superabundance [15], [16]. For most systems, we still have very limited understanding of how such outbreak-induced resource pulses affect the dynamics of consumer communities.

Saproxylic insects are one group of organisms that may experience particularly dramatic increases in resource availability following outbreaks. Saproxylic insects are, per definition, “dependent, during some part of their life cycle, upon the dead or dying wood of moribund or dead trees, or upon wood-inhabiting fungi or the presence of other saproxylics” [17]. Thus, since outbreaks kill or damage a large number of trees within a short time, they may provide saproxylic insects with resource pulses in the form of massive amounts of dead or dying woody material. Accordingly, saproxylic insects could be expected to increase rapidly in abundance after outbreaks. Indeed, it is well documented that saproxylic insects respond numerically to large-scale forest mortality due to other disturbances, like fire [18]–[20] and wind [21]–[23], and recently this has also been reported after outbreaks by tree-killing bark beetle in central Europe [24]. However, the magnitude of such responses is not necessarily a simple function of resource input. One important limiting factor may be the intimate relationship between saproxylic insects and the stage of decay of dead wood. A given saproxylic species can usually exploit dead wood only in a specific stage of decay. Thus, at any point in time after an outbreak, only a subset of the saproxylic community will be able to utilize the dead wood resource pulse. Moreover, the decay stage a saproxylic species can utilize is often relatively short in duration, lasting no more than a few years [25]–[27]. This is likely to lead to satiation effects, where a species is able to colonize and exploit only a small fraction of the dead wood generated by an outbreak within the time period when the wood represents a usable resource. Consequently, numerical responses to outbreaks both in individual species and the saproxylic community as a whole may in many cases be relatively limited in magnitude, compared to the magnitude of dead wood input. It should also be kept in mind that insect populations may be naturally limited or regulated by numerous other abiotic and biotic factors beyond the abundance of their resources, like climate and natural enemies [28].

Saproxylics are strongly affected by the successional decay of wood, but they also contribute to determining the speed of this decay process. When saproxylic insects penetrate the surface of dead wood, they contribute to its physical degradation, improve aeration of the wood and facilitate the entry of decomposing fungi and bacteria [27], [29]. This can increase the rate of decay of the wood [30], [31]. Thus, the ability of saproxylic insects to respond numerically to the dead wood left by an insect outbreak may affect the speed with which the dead trees are decomposed, and, ultimately, the resilience of the whole forest ecosystem. Understanding the numerical responses of saproxylic insects is, therefore, an important component of understanding the post-outbreak dynamics of forest ecosystems. However, most studies of how saproxylics respond to large-scale forest disturbances have dealt with fire and wind, whose effects are not necessarily directly comparable to those of insect outbreaks. Wind mainly produces lying dead trees, which tend to have high moisture content, while outbreaks leave mostly standing dead trees, which are prone to desiccation. This difference is expected to cause contrasting successional trajectories in the saproxylic community, particularly in the early stages of succession [27]. Meanwhile, trees killed by fire favour a specialized guild of fire-adapted saproxylics [19]. As far as we are aware, the bark beetle outbreaks in the Bavarian Forest National Park in Germany represent the only case where the responses of saproxylic insects to insect outbreaks have been studied so far [24], [32], [33]. Thus, the knowledge of this subject is still very limited.

**Figure 1:**
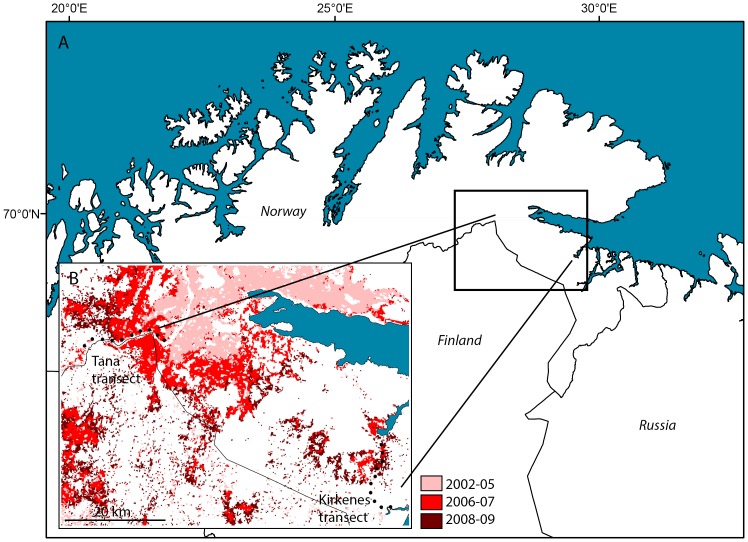
Map of Finnmark County (A) with the study region in Varanger inset (B). Red areas represent birch forest which experienced severe defoliation during the most recent moth outbreak. Different shades indicate the main years of defoliation for different areas (see key). The sampling stations of the two transects are indicated by black dots.

In the present study, we examine the responses of saproxylic beetles to a massive outbreak of the two geometrid moths (Lepidoptera: Geometridae) *Epirrita autumnata* (Bkh.) (autumnal moth) and *Operophtera brumata* (L.) (winter moth) that occurred in the mountain birch (*Betula pubescens* ssp. *czerepanovii* Orlova) forest of the Varanger region in northern Norway (Fig. 1A) during 2002–2009. Mortality of birch trees was high throughout this region and reached more than 95% locally [14], [34]. Thereby, the availability of dead wood for saproxylic insects was elevated far above normal levels across a very large area. In order to assess the short-term responses of the saproxylic beetle community in Varanger to this massive dead wood resource pulse, we used window traps to sample beetles in 2011 and 2012, both in severely damaged forest and undamaged forest at the outskirts of the outbreak area. Non-saproxylic beetles were also included in the study, in order to provide a contrast group that is not expected to benefit from increased availability of dead wood. By comparing beetle counts between damaged and undamaged forest, we address the following specific questions:

1. What is the magnitude of the short-term numerical responses of saproxylic beetles (both as individual species and as a community) to the superabundance of dead wood resources generated by the moth outbreak?
2. Are the numerical responses of saproxylic beetles weak compared to the magnitude of dead wood resource input, suggesting that important limiting factors act to constrain beetle responses?
3. Have numerical responses occurred only in saproxylic beetle species that are associated with dead wood in a particular stage of decay, suggesting decay-stage-preferences as a limiting factor for beetle responses?
4. Has the outbreak caused changes in saproxylic beetle community structure (i.e. the relative abundances of different beetle species), as would be expected if only a subset of the species in the community have responded numerically to the outbreak (cf. question 3)? If so, what is the magnitude of this change in community structure?

## Materials and Methods

### Ethics statement

No specific permissions were required to access any of the sampling locations or deploy any of the sampling methodologies described for this study. Three beetle species that are included on the Norwegian red list [35] were incidentally caught (see below for description of the trapping method) in small numbers during the study, namely *Atheta taxiceroides* (IUCN category: Near threatened), *Denticollis borealis* (IUCN category: Vulnerable) and *Enicmus lundbladi* (IUCN category: Vulnerable). This has been reported to the Norwegain Biodiversity Information Centre (http://www.biodiversity.no/). Table S1 in Appendix S1 gives the number of individuals caught.

### Study region

The Varanger region is located at approximately 70°N, 29°E, in the north-eastern part of Finnmark county, northern Norway (Fig. 1A). Mean temperatures for January and July are –12.2 and 12.3°C, respectively, and annual precipitation is 400–500 mm [Rustefjelbma meteorological station (70°23′55″N, 28°11′36″E), 1961–2000 normal period]. The forest in the region is strongly dominated by mountain birch, although aspen (*Populus tremula*) and Scots pine (*Pinus sylvestris*) also occur patchily. Outbreaks by *E. autumnata* and *O. brumata* are the main natural disturbance factors in the north-Fennoscandian mountain birch ecosystem and occur approximately every 10 years [36]–[38]. Outbreaks vary in intensity, but sometimes cause severe defoliation and high mortality rates of mountain birch across thousands of square km. The duration and severity of the last outbreak cycle was historically unprecedented, and it has been estimated that approximately 10 000 square km of birch forest in northern Fennoscandia was affected by severe defoliation at least once during the period 2002–2008 [34]. The Varanger region was particularly strongly affected due to consecutive severe outbreaks by *E. autumnata* (2002–2004) and *O. brumata* (2005–2009).

### Study design and sampling

In spring 2011, two transects were established in areas with slightly different defoliation histories, namely Tana (70°03′ N, 27°45′ E. Defoliated mainly during 2006–2007) and Kirkenes (69°46′ N, 29°20′ E. Defoliated mainly during 2007–2009) (Fig. 1B). Both transects were roughly linear and consisted of 10 sampling stations, interspaced by approximately two km. The first stations in each transect were located deep within forest which had suffered severe mortality due to the outbreak. Both transects then ran towards forest which was located at the outskirts of the outbreak area and had only experienced negligible defoliation (Fig. 1B). Fig S1 in Appendix S1 shows photographs from the individual sampling stations.

To assess the level of forest damage inflicted by the outbreak at the individual sampling stations, we established two 50 m survey lines at each station. The lines started at the centre-point of the station and ran towards the north and south, respectively. Every 7 m along the lines, we selected the closest birch tree with at least one stem (standing or fallen) of more than 1.3 m in height. The damage of the three thickest stems (above a minimum height of 1.3 m) within each selected tree was visually scored according to the criteria in table 1. These registrations were only conducted in 2011, since we had no reason to believe that the level of forest damage would change importantly between the two study-years (i.e. since the outbreak had ended in 2009).

**Table 1:** Classification system used to score the damage of birch stems.

Damage category	Definition	Damage score
Living. Undamaged	Stem with no apparent loss of foliage	1
Living. Lightly damaged	Stem with >50% of foliage reaming compared to healthy stem	2
Living. Severely damaged	Stem with <50% of foliage reaming compared to healthy stem	3
Dead	Dead stem without foliage	4

**Figure 2:**
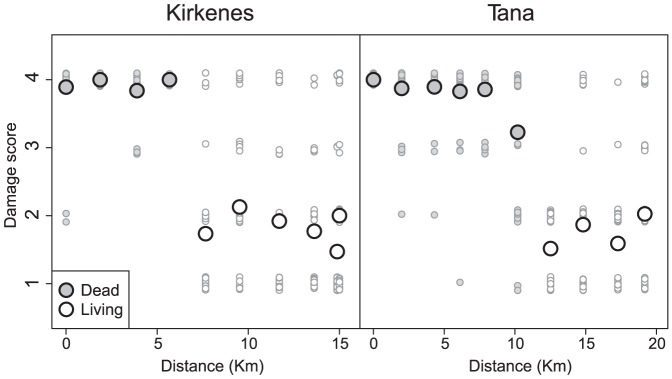
Station-specific damage scores of birch stems along the two sampling transects. The X axes represent the airline distances from the first sampling station in each transect. Large circles represent mean damage scores for each station, while small circles represent scores for individual stems. A small amount of noise has been added to the stem-specific scores to help distinguish individual datapoints. See table 1 for explanation of damage scores.

Inspection of damage scores for the individual sampling stations showed that the damage-level of the forest shifted abruptly along both transects (Fig. 2). Station 1–4 in Kirkenes and 1–5 in Tana had almost exclusively dead stems, and were therefore designated as “dead” stations. In contrast, at station 5–10 in Kirkenes and 7–10 in Tana, the great majority of stems were alive and either undamaged or with light loss of foliage. This is representative for the situation in a normal, healthy mountain birch forest. These stations were therefore designated as “living”. Station 6 in Tana had a preponderance of dead stems, but also many living ones. The station was designated as dead, since its mean damage score fell closest to the dead stations. The simple dichotomy of dead and living stations was used to represent forest damage in all subsequent analyses. Levels of forest damage at the individual stations were representative for the surrounding landscape (Fig. 1B. [34]). Thus, within a transect, dead and living stations were located within continuous belts of severely damaged and healthy forest, respectively.

Sampling of beetles was conducted by means of large window traps, with two crossed 40×60 cm window panes [39]. All traps were mounted between two adjacent birch stems, with the upper edge of the window panes approximately 1.5–2 m above the ground (Fig. S1 in Appendix S1). We used three traps per sampling station (for a total of 60 traps across the two transects). One trap was located at the centre point of the station, while the other two were located approximately 50 m to the north and south, respectively. In both 2011 and 2012, the traps were mounted in early June and remained operative for approximately two months until early August (thereby covering the peak of insect activity in our high-latitude study system).

All beetle individuals were identified to species level by an expert. Beetle species were thereafter classified according to their degree of association with dead wood, i.e. obligate saproxylic, facultative saproxylic or non-saproxylic, hereafter collectively referred to as dead wood association (DWA) groups. All species were also classified according to larval trophic guild, i.e. predators, fungivores, herbivores, wood-feeding, several (species belonging to more than one guild) or others (guilds that were rare in this study, i.e. scavengers, coprophages and detritus-feeders). Finally, saproxylic species were classified according to host-tree association i.e. associated with birch, not associated with birch and unknown host-tree association. The classification was based mainly on Dahlberg and Stokland [40] and Böhme [41], but additional information was obtained from a number of other sources. Table S1 in Appendix S1 gives a complete list of the sources used.

### Statistical analyses

The goal of our analyses was to address the outlined questions about the numerical responses of saproxylic beetles in the study region to the changes in dead wood availability caused by the moth outbreak. The availability of dead wood was represented by our classification of forest damage, i.e. living forest (normal background levels of dead wood) and dead forest (greatly elevated availability of dead wood owing the moth outbreak). Since there was an abrupt shift from living to dead forest along the transects, we also expected that beetle communities would show rather sharp (i.e. non-gradual) spatial changes when moving from one forest type to the other. Therefore, we assumed that spatial changes in the beetle community between living and dead forest were adequately modelled by a categorical living-dead contrast. The possibility of additional spatial patterns in the data was assessed by plotting station-specific beetle counts as well as topographical parameters along the transects.

Forest damage (dead or living) was the focal predictor variable in all analyses. In addition, all models included the categorical predictors year (2011 or 2012) and location (Kirkenes or Tana), to account for variation in beetle communities between study years and transects. Within each year, beetle counts were pooled across the entire trapping season and the three individual traps within each station, resulting in a single datapoint per station, per year. All analyses were conducted separately for the three DWA groups, owing to their different degrees of association with dead wood. For the two saproxylic groups, the analyses only included species which are known to be associated with birch and which could therefore be expected to respond numerically to the dead birch wood left by the outbreak.

We performed quantitatively oriented univariate analyses to assess the magnitude of the numerical responses of saproxylic beetles to the dead wood generated by the moth outbreak. First, we assessed whether the total abundances of the different DWA groups (pooled abundance across all species within each group) differed between dead and living forest. This was done by fitting log-linear models with the station-specific individual-counts of each DWA group as response variables, and forest damage, year, location and their two-way interactions as predictors. All models employed quasi-likelihood correction for overdispersion. We used the Akaike Information Criterion, corrected for small sample size and overdispersion (QAIC_c_), to select the best model for each DWA group. The minimal models included only the main effect of the focal predictor forest damage.

We also investigated if the proportion that each DWA group constituted, out of the total number of individuals across all groups, differed between dead and living forest. This was done by fitting logistic regression models with the station-specific proportions of each DWA group as response variables. We employed the same predictor variables and model selection strategy for this analysis as for the analysis of the total counts of DWA groups (above).

To assess numerical responses to dead wood availability in the most important saproxylic beetle species, we fitted log-linear models to the station-specific counts of all species which were judged to be sufficiently abundant for statistical modelling (total count >80 individuals). The predictor variables and model selection strategy for these species-specific models were the same as for the models fitted to the total counts of the DWA groups (above).

Finally, we used multivariate methods to investigate how beetle community structure varied between living and dead forest. In order to assess overall community-responses to the outbreak, the multivariate models were run not only for the three DWA groups, but also collectively for all species in the data. All beetle counts were Hellinger transformed before multivariate analysis [42].

We performed redundancy analysis (RDA) (rda function in the R package vegan) to allow visualization and significance-testing of the effects of forest damage, year and location on beetle community structure. We assumed additive effects of the three predictors, since inclusion of interactions did not change the conclusions from the analysis. The significance of the marginal effects of the predictors (i.e. effects after accounting for all other predictors in the models) was assessed with permutational ANOVA (anova.cca function in the R package vegan) with a maximum of 9999 permutations. Site scores from the RDAs were displayed on biplots in order to investigate how the beetle communities of the individual sampling stations were organized with respect to the predictor variables.

Variance partitioning (varpart function in the R package vegan) was used to estimate how much of the variance in beetle community structure was explained by the focal predictor forest damage, as well as year and location. The results of the variance partitioning were visualised with venn diagrams, fitted with the venneuler function in the R package with the same name. Negative variance fractions were treated as zeros.

## Results

### Overall beetle community-structure

A total of 5880 beetle individuals, belonging to 207 different species, were caught during the study (Table 2. Table S1 in Appendix S1 provides a complete species list). Obligate-, facultative- and non-saproxylic species accounted for 28.2%, 12.5% and 58.1% of the individuals and 25.1%, 22.7% and 43.5% of the species, respectively. The degree of association with dead wood is unknown for 1.2% of the individuals and 8.7% of the species. Predators and fungivores were the most abundant and species-rich trophic guilds among obligate saproxylic species, while predators and herbivores were most abundant and species-rich among non-saproxylic species (Table 2). Predators also constituted the highest number of individuals and species among facultative saproxylics. Facultative saproxylics also included many individuals and species that were fungivores or belonged to several trophic guilds, as well as many individuals with unknown trophic guild (most of which belonged to the species *Liotrichus affinis*). Most of the obligate saproxylic individuals (85.5%) and species (78.8%) caught were associated with birch. For facultative saproxylics, most of the individuals (70.4%) and roughly half of the species (48.9%) were birch associated.

**Table 2:** The total number of individuals and species (in brackets) caught during the study for each DWA group and larval trophic guild.

	Dead wood association (DWA) group				
Trophic guild	Obligate sx.	Facultative sx.	Non-sx.	Unknown	Sum
Predator	597 [15]	215 [25]	1859 [44]	29 [9]	2700 [93]
Fungivore	820 [20]	63 [14]	65 [16]	36 [6]	984 [56]
Herbivore	-	-	1251 [13]	-	1251 [13]
Wood-feeding	71 [5]	-	-	-	71 [5]
Several	167 [11]	33 [5]	1 [1]	-	201 [17]
Other	-	-	86 [9]	-	86 [9]
Unknown	4 [1]	422 [3]	155 [7]	6 [3]	587 [14]
Sum	1659 [52]	733 [47]	3417 [90]	71 [18]	5880 [207]

### Effects of dead wood availability on the total abundances and proportions of DWA groups

**Figure 3:**
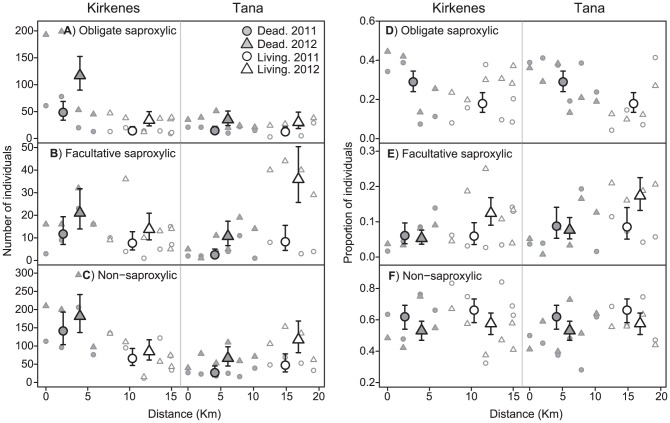
Variation in abundances and proportions of DWA groups along the two sampling transects. Large symbols: predicted abundances from log-linear models (A–C) and proportions from logistic models (D–F) in dead and living forest in Kirkenes and Tana in 2011 and 2012. Error bars represent 95% confidence intervals. In cases where year effects were not supported by the model selection, only a single predicted value for each damage × location combination is presented, representing the overall estimate across 2011 and 2012. Small symbols: original datapoints, i.e. the observed station-specific counts (A–C) and proportions (D–F) of obligate- facultative- and non-saproxylic beetle species in Kirkenes and Tana in 2011 and 2012, plotted against the airline distance from the first station in each transect.

Outputs from the log-linear models (see table S2 and S3 in Appendix S1 for model selection and parameter estimates) for all three DWA groups were heavily influenced by large-scale spatial gradients in beetle counts along the transects (Fig. 3A–C). These gradients did not appear to have a consistent relationship with forest damage (i.e. their direction with respect to forest damage shifted between the two transects). Nevertheless, the results of the modelling were consistent with our expectation of a positive numerical response to increased dead wood availability for obligate saproxylic species, but not for facultative saproxylic species. In particular, the predicted abundances of all three DWA groups were higher in dead than in living forest in Kirkenes. However, this effect was most pronounced for obligate saproxylics (Fig. 3A), which had roughly 3.5 times higher predicted abundance in dead forest in this transect, while the predicted abundances of facultative- and non-saproxylics were only about 1.5 and 2 times higher, respectively, in dead forest (Fig. 3B, C). In Tana, the patterns for facultative- and non-saproxylics showed a reversal with respect to forest damage, with predicted abundances that were roughly one third and half as high in dead as in living forest (Fig. 3B, C). Meanwhile, the predicted abundance of obligate saproxylics was very similar for the two forest types (Fig. 3A). These patterns were consistent across both study-years (i.e. model selection did not support damage × year interactions for any DWA group), although the overall abundances of all three groups were considerably higher in 2012. Thus, when the three DWA groups were contrasted, it would appear that obligate saproxylics were favoured by dead forest. This impression was supported by logistic modelling of the relative proportions of the DWA groups (see table S3 and S4 in Appendix S1 for model selection and parameter estimates). The predicted proportion of obligate saproxylic individuals was about 10% higher in dead than in living forest (odds ratio: 1.87. 95% CI: 1.22–2.87. Fig. 3D). Meanwhile, there was a roughly 4% decrease in the predicted proportion of non-saproxylic individuals in this forest type (odds ratio: 0.83. 95% CI: 0.59–1.16. Fig. 3E). These patterns were consistent both across locations and years (i.e. model selection did not support damage × year or damage × location interactions for these two groups). The predicted proportion of facultative saproxylic individuals was very similar in living and dead forest in 2011(odds ratio: 1.03. 95% CI: 0.51–2.07. Fig. 3F). However, the model selection supported the damage × year interaction for this DWA group, causing the predicted proportion of facultative saproxylics to be almost 10% lower in dead forest in 2012 (odds ratio: 0.39. 95% CI: 0.25–0.62. Fig. 3F).

### Effects of dead wood availability on individual beetle species

**Figure 4:**
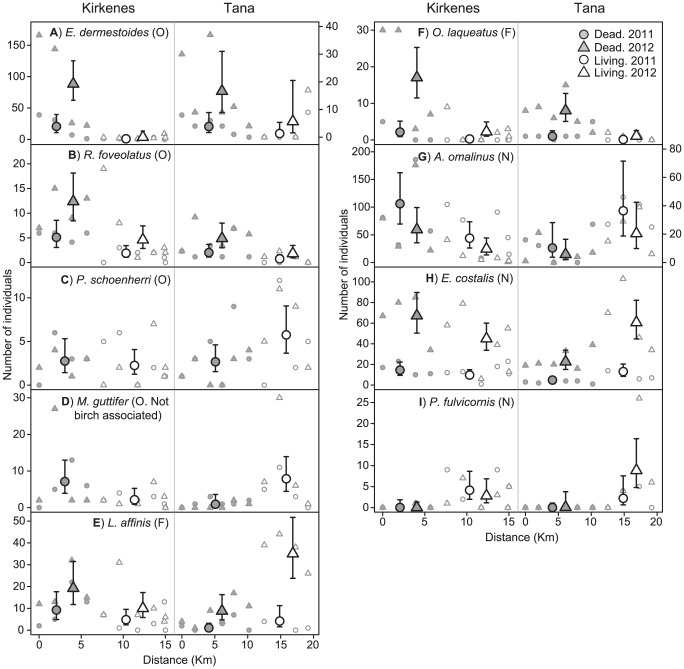
Variation in abundances of individual beetles species along the two sampling transects. Large symbols: predicted abundances from log-linear models in dead and living forest in Kirkenes and Tana in 2011 and 2012. Error bars represent 95% confidence intervals. In cases where year effects were not supported by the model selection, only a single predicted value for each damage × location combination is presented, representing the overall estimate across 2011 and 2012. Small symbols: original datapoints, i.e. the observed station-specific counts of the beetle species in Kirkenes and Tana in 2011 and 2012, plotted against the airline distance from the first station in each transect. The degree of dead wood association for each species is given in parenthesis behind its name. O =  Obligate saproxylic. F =  Facultative saproxylic. N =  Non-saproxylic. All saproxylic species except *Malthodes guttifer* are known to be associated with birch. Note different scales on the Y axis in the plots for Kirkenes and Tana for *Elateroides dermestoides* (A) and *Anthophagus omalinus* (G).

Three birch associated obligate saproxylic, one birch associated facultative saproxylic and four non-saproxylic species were judged to be abundant enough for individual log-linear modelling (Fig. 4. See table S5 and S6 in Appendix S1 for model selection and parameter estimates). These species collectively constituted 69.1% of all individuals in the data, 63.0% of all birch associated obligate saproxylic individuals, 81.2% of all birch associated facultative saproxylic individuals and 80.5% of all non-saproxylic individuals. The obligate saproxylic *Malthodes guttifer* was also abundant enough for modelling, but not associated with birch. Nevertheless, we analysed this species for the sake of comparison with the other saproxylics.

Two of the birch associated obligate saproxylic species, namely *Elateroides dermestoides* (Fig. 4A) and *Rabocerus foveolatus* (Fig. 4B), showed evidence for a positive numerical response to dead wood availability, with higher predicted abundances in dead than living forest in both Kirkenes and Tana (*E. dermestoides*: roughly 25 and 3 times more abundant in dead forest in Kirkenes and Tana, respectively. *R. foveolatus*: roughly 2.5 times more abundant in dead forest in both transects). Curiously, the non-saproxylic species *Oxytelus laqueatus* (Fig. 4F) showed a similar pattern (roughly 7.5 times more abundant in dead forest in both transects). The obligate saproxylic *Podistra schoenherri* (Fig. 4C) and the facultative saproxylic *L. affinis* (Fig. 4E) (both birch associated) did not appear to respond consistently to dead wood availability, with predicted abundances being higher in dead forest in Kirkenes and lower in this forest type in Tana. A similar reversal of predicted abundance patterns between transects were evident for the not birch associated obligate saproxylic species *M. guttifer* (Fig. 4D) and the non-saproxylic species *Anthophagus omalinus* (Fig. 4G) and *Eanus costalis* (Fig. 4H). Finally, the non-saproxylic species *Polydrusus fulvicornis* (Fig. 4I) was almost absent in dead forest but relatively abundant in living forest in both transects.

### Effects of dead wood availability on beetle community structure

**Figure 5:**
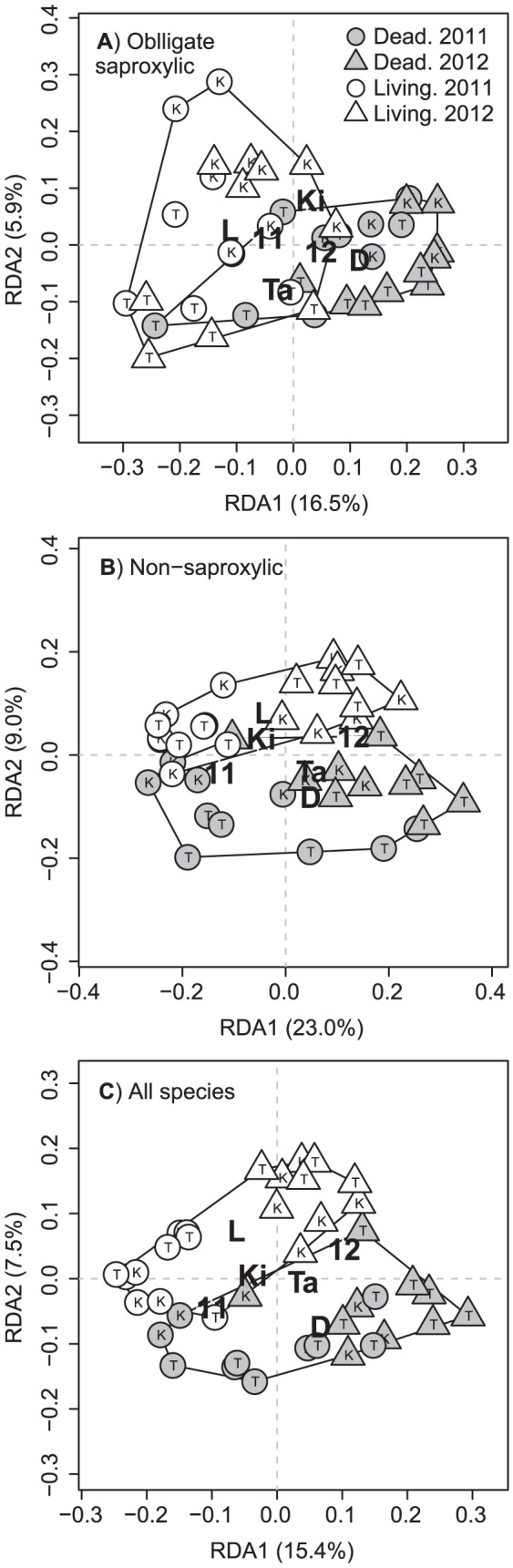
Biplots relating the predictors forest damage, location and year to variation in beetle community structure. A: Obligate saproxylic species. B: Non-saproxylic species. C: All beetle species collectively. Circles and triangles represent sites scores (corresponding to individuals sampling stations) from the RDAs. The letters on the symbols indicate the transect that each station belongs to. K =  Kirkenes. T =  Tana. Centroids for the levels of the three predictor variables are indicated by large letters on the plots. Forest damage: D =  Dead. L =  living. Location: Ki =  Kirkenes. Ta =  Tana. Year: 11 = 2011. 12 = 2012.The two polygons in each plot surround all stations belonging to dead and living forest. Note that the site scores are shown as principal coordinates [65], implying that the distances between points are two-dimensional approximations of the Hellinger distances between them. The percentage of the total variation that is accounted for by the RDA axes is given in the axis labels.

Forest damage explained none of the variance in community structure for facultative saproxylics (see below) and we therefore had no reason to expect that damage would yield significant effects in a RDA for this DWA group. Accordingly, we did not conduct RDA for facultative saproxylics. RDA site scores for living and dead forest tended to be separated in biplots for both obligate- and non-saproxylic species, suggesting that these DWA groups showed consistent differences in community structure between the two forest types (Fig. 5). This separation was quite distinct for non-saproxylic species (Fig. 5B), but less clear for obligate saproxylics (Fig. 5A), with considerable overlap between living and dead stations for the latter group. Nevertheless, the effect of forest damage was highly significant in permutation-based ANOVAs for both DWA groups (*P* = 0.005 in both cases). When all beetle species were considered collectively, the separation between living and dead stations became even clearer, with hardly any overlap between the two forest types (Fig. 5C). Also in this case, permutation-based ANOVA yielded a highly significant effect of forest damage (*P* = 0.005). The degree of separation between site scores for Kirkenes and Tana and 2011 and 2012 was variable among the species groups, but all permutation-based ANOVA tests for location and year were significant at *P* = 0.030 or lower.

**Figure 6:**
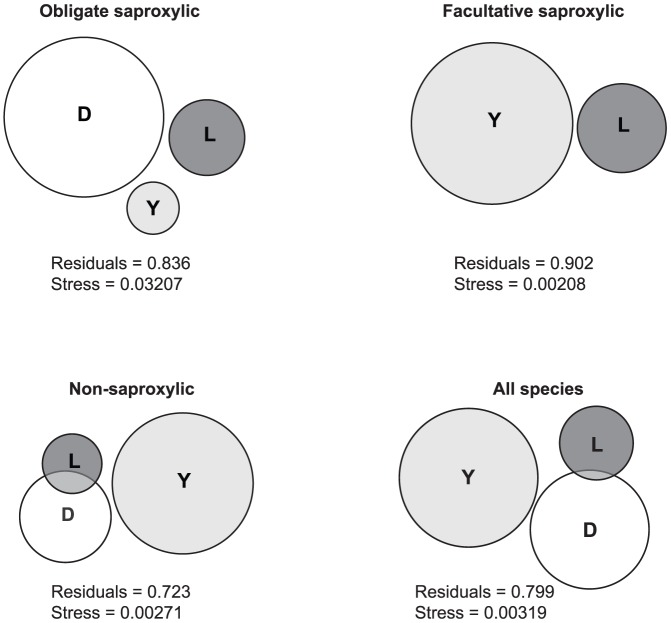
Contributions of forest damage, location and year to explaining variance in beetle community structure. D =  damage, L =  location and Y =  year. The amount of variance explained by each predictor is represented by the relative size of the circles in the venn diagrams. Overlapping areas indicate variance that is jointly explained by two of the predictors, while non-overlapping areas indicate variance that is uniquely explained by a single predictor. The residuals represent the proportion of the total variance in the beetle data that cannot be explained by the predictors. The values of the venneuler stress statistic [66] are below the 0.01 threshold (s._01_ = 0.056) in all cases, indicating that there is a good match (i.e. fit) between the venn diagrams and the actual variance components (Table 3).

Forest damage, location and year collectively explained 16.4% of the variance in beetle community structure for obligate saproxylic species, 27.7% for non-saproxylic species and 20.1% for all species (Table 3, Fig. 6). For facultative saproxylics, damage failed to account for any variance, while location and year collectively explained 9.8%. In all cases, there was little overlap between the variance explained by the individual predictors, so that their total and unique contributions were largely similar. We therefore report only total contributions below. Damage was the most important predictor for obligate saproxylics, accounting for 11.1% of the variance. Year was most important for facultative saproxylics, accounting for 7.4%. Year was also most important for non-saproxylics, accounting for 17.7%, while damage made a comparatively modest contribution of 7.6%. As expected from the results for the individual DWA groups, the relative contributions of year and damage became more even when all beetle species were considered collectively, with year explaining 10.2% and damage explaining 7.3%. Location made only a small contribution for all groups, explaining less than 4.0% of the variance in all cases.

**Table 3:** Variance partitioning table showing how the predictor variables forest damage, location and year contribute to explaining the variance in beetle community structure for obligate-, facultative- and non-saproxylic (sx.) beetles, and for all beetle species collectively.

	Df	Adjusted *R^2^*			
Predictors		Obligate sx.	Facultative sx.	Non-sx.	All species
Damage	1	0.111	0	0.076	0.073
Location	1	0.038	0.024	0.035	0.030
Year	1	0.016	0.074	0.177	0.102
Damage + Location	2	0.149	0.024	0.100	0.099
Damage + Year	2	0.126	0.074	0.253	0.175
Location + Year	2	0.054	0.098	0.212	0.132
All	3	0.164	0.098	0.277	0.201
*Individual fractions*					
Damage| Location + Year	1	0.111	0	0.063	0.069
Location | Damage + Year	1	0.038	0.024	0.022	0.026
Year | Damage + Location	1	0.016	0.074	0.177	0.102
Residuals		0.836	0.902	0.723	0.799

## Discussion

Insect outbreaks can rapidly generate more dead woody material than any other ecosystem process (with the possible exception of wind and fire), but little is known of how saproxylic organisms respond to these disturbance events, despite the fact that saproxylics are intimately dependent on dead wood and play an important role in the ecosystem service of decomposition [43], [44]. In this study we have contributed to filling this knowledge-gap by characterizing short-term changes in the abundance and community structure of saproxylic beetles following a severe moth outbreak in sub-arctic birch forest.

We expected that the outbreak would generate a dead wood resource pulse and thereby trigger positive numerical responses in the saproxylic beetle community. Importantly, we also expected that such responses would be limited to a subset of the community, owing to the ability of given saproxylic species to exploit only certain dead wood decay-stages, and that increases in responding species would be constrained by limitations to the amount of dead wood that can be colonized within a given amount of time (i.e. satiation effects). Our results appear to match these expectations. By contrasting the overall abundances of obligate-, facultative- and non-saproxylic species, we did indeed find evidence for increased abundance of obligate saproxylics in outbreak-affected forest. However, this increase corresponded only to a roughly 10% higher proportion of obligate saproxylic individuals in dead forest, while the increase in the dead wood resource may have been as much as hundredfold.

These findings may suggest that the saproxylic beetle community in the study region has a limited ability to respond to increased availability of dead wood. Our results for individual beetle species suggest species-specific decay-stage-preferences as one potential reason for this limitation. Most of the numerical response of obligate saproxylics was driven by the two species *E. dermestoides* and *R. foveolatus*. Both species exploit recently dead or dying trees and have therefore probably enjoyed increased resource-availability during the 3–5 years separating the conclusion of the outbreak and our study. Meanwhile, the third birch associated obligate saproxylic species that was analysed (*P. schoenherri*) showed no response to the outbreak. This species utilizes dead wood in more advanced stages of decay and is thereby unlikely to have benefitted from the outbreak so far. Thus, our findings match the prediction that species-specific decay-stage-preferences will allow only a subset of the saproxylic beetle community to respond to an outbreak at any given time. Unfortunately, the knowledge of decay-stage-preferences for most species in our data is too limited to allow more rigorous evaluation of this hypothesis.

Our study gives no direct indication of whether the numerical response of the saproxylic beetle community is constrained by satiation effects. However, this seems likely to be the case. Within a given area, several decades can pass between moth outbreaks that cause substantial forest mortality. It is therefore unlikely that saproxylic beetles in the study region are specifically adapted to efficiently exploit the extreme amounts of dead wood that outbreaks generate. Satiation effects could result e.g. from limitations to the amount of dead wood that adult beetles manage to colonize and reproduce in within a given time, including the number of eggs that they have available. Reproductive rates could be further constrained by the cold climate of the study region, which may prolong larval development of the decomposing insect community [45]. Such limitations on reproductive output may be decisive for the magnitude of numerical responses to the outbreak, since these responses are probably driven mainly by reproduction. Behavioural aggregation of beetles to the dead forest seems unlikely to be important, since the outbreak area is very large (Fig. 1B) and a massive number of individuals would have to be drawn in from adjacent forest areas in order to raise densities appreciably. Moreover, the spatial extent of the outbreak area is probably far larger than the dispersal capacity of most beetle species in the data. Notably though, since the level of forest damage inflicted by the outbreak is spatially heterogeneous, aggregation on smaller spatial scale may affect local density patterns within the outbreak area.

Climatic constraints on beetle diversity in our sub-arctic study region may also help explain the seemingly weak overall response of the saproxylic beetle community. In total, we encountered 52 obligate- and 47 facultative saproxylic species. This is considerably less than what is typically reported from studies with comparable sampling efforts in more southerly deciduous forests [46]–[48] Thus, many species which may have responded to an outbreak further south may simply be absent from our system. Some support for this suggestion is provided by the results of Wermelinger *et al.* [21], who studied short-term responses of saproxylic beetles to a large-scale windthrow event in Switzerland. Within 6 years of the windthrow, they observed much stronger increases in the abundance of saproxylic beetles than we did in the present study. This was largely due to species in the families Cerambycidae and Buprestidae and the subfamily Scolytinae (all of which contain birch associated species). These three groups were either absent (Buprestidae) or very rare (Cerambycidae: 1 species, 11 individuals. Scolytinae: 4 species, 16 individuals) in our data, presumably because our study region lies beyond the northern distributional limit of most of their species [25]. Our system may thereby lack many species that could potentially have responded numerically to a dead wood resource pulse. Another factor to consider is that our study region represents the northern distributional limit for most of the beetle species we encountered. Many species may thus be close to their tolerance limits for abiotic factors, like temperature, and could therefore be highly sensitive to stochastic year-to-year environmental variation [49]. This might also impair their ability to respond effectively to increases in resource availability.

It should be noted that our interpretation of window trap catches in terms of beetle numerical responses has some potential pitfalls. First, local sun exposure can potentially affect beetle activity levels, so that beetle counts are inflated in exposed (i.e. warmer) conditions. Sun exposure may also affect actual beetle abundance, since many saproxylic species have preferences for exposed or shaded conditions [50], [51]. In our case, stations in dead forest (without foliage in the canopy) may have been more sun exposed than stations in living forest (with an intact canopy). However, total beetle counts were not consistently higher in dead forest (Fig. 3), suggesting that sun exposure did not inflate beetle counts in our case. Similarly, Kaila *et al*. [39] and Sverdrup-Thygeson and Ims [52] had similar total beetle counts in exposed and shaded conditions. With respect to beetle habitat preferences, we are not aware that any of the most abundant species in our data (saproxylic or non-saproxylic) are considered to have strong preferences relating to sun exposure. Thus, we have no reason to expect that sun exposure was an important driver of the main abundance patterns in our study. Also, we should emphasize that even healthy mountain birch forest is quite open, with mostly small and well-spaced trees (This is well illustrated by Fig. S1 in Appendix S1). Thus, the degree of sun exposure may not have differed radically between stations in living and dead forest. Second, saproxylic beetles may respond to variation in dead wood availability only on certain spatial scales, and window traps may fail to detect these responses if the volume of dead wood around the traps has not been quantified at the relevant scale [53]–[55]. However, in our case, the volume of dead wood was extremely high not only in the immediate vicinity of the traps, but also throughout the outbreak area as a whole, i.e. on a scale of thousands of hectares around the traps. Thus, in effect, our design captured elevated availabilities of dead wood on a wide range of spatial scales, ranging from locally around the traps and up to the landscape level. Thereby, we should have been able to detect beetle responses to the outbreak, regardless of whether beetles responded to dead wood availability on small or large spatial scales. Third, dispersal-driven “spillover” of saproxylic beetles from dead to living forest may have elevated beetles densities in living forest. This would invalidate our conclusion about weak numerical responses to the outbreak based on comparing beetle catches between the two forest types. However, such spillover would probably have been manifested as a gradual decline in saproxylic beetle counts when moving progressively further away from dead forest. This did not appear to be the case in our data. For beetle counts to be uniformly elevated throughout living forest there would have to be high rates of dispersal over distances of at least 10 km (the approximate distance between the last living and dead stations in both transects). This seems improbable, although the dispersal capacity of the studied beetles is too poorly known to rule out the possibility either. Thus, in summary, although our results must be cautiously interpreted, we still consider it to be a reasonable conclusion that saproxylic beetles have so far responded weakly to the dead wood left by the outbreak.

If the ability of the saproxylic beetle community to respond numerically to dead wood resource pulses is indeed limited, this could have important implications for the decomposition of trees killed by the outbreak. Saproxylic beetles contribute to wood decay by direct consumption and physical degradation of woody material, and by facilitating the entry and activity of decomposing fungi and bacteria [27], [29]–[31], [43], [44]. However, if saproxylic beetles respond weakly to dead wood input, such decay processes may so far have played a minor role in decomposing the trees killed by the outbreak. Thereby, the decay of these trees may be retarded compared to dead wood in healthy mountain birch forest. This possibility must be explored in future studies, since we did not quantify the decay of dead stems along the transects. Nearly all stems were still standing in 2012 (pers. obs. by the authors), but this tells us little about decay rates, since birch stems have more than 80% probability of remaining standing throughout the first decade after their death (Mäkinen *et al.* [56] studying *B. pubescens* and *B. pendula* in southern and central Finland). Studies that contrast decay and insect-utilization of dead trunks between outbreak areas and healthy forest would be instructive, as would studies of fungal and other microbial decomposer communities. Penetration of bark by saproxylic invertebrates can be important for the colonization of dead trunks by bacteria and fungi [29], and weak numerical responses of saproxylic beetles to outbreaks may thereby retard colonization or alter community-composition in these microbial decomposers. Alternatively, the relative importance of microbial decomposition may increase in the absence of strong numerical responses from saproxylic beetles. Studies addressing these questions would be of great interest for understanding post-outbreak decomposition dynamics.

The indication of limitations in the short-term numerical response from the saproxylic beetle community to increased dead wood amounts is also relevant in a management perspective. Several studies, especially in Northern Europe, have addressed the negative effects the decrease in background levels of dead wood in managed boreal forest have on the saproxylic beetle communities [52], [57], [58]. In order to alleviate these effects, new management guidelines and restoration efforts are suggested to increase the dead wood amounts [53], [59]–[61]. In this process, a firm understanding of the responses of saproxylic beetles across a wide span of spatial and temporal scales, and in different forest ecosystems, is vital.

The separation of sites in living and dead forest in the RDA for obligate saproxylics indicates that the outbreak has induced changes in community structure (i.e. changes in the relative abundances of different species) among saproxylic beetles. This fits well with our expectation that only some saproxylic species should respond numerically to the outbreak, and the empirical observation of species-specific response patterns. A more unexpected finding is the clearer separation (i.e. larger community changes) of dead and living sites for non-saproxylic species. This may suggest that there has been greater variation in species responses to the outbreak among non-saproxylics than obligate saproxylics. This seems plausible, since the non-saproxylic group contains more species and may therefore encompass a higher diversity of biological traits (e.g. microhabitat requirements and feeding specialisations). It is noteworthy in this respect that there were clear examples of both positive (i.e. *O. laqueatus*) and negative (i.e. *P. fulvicornis*) responses to the outbreak among the individually analysed non-saproxylic species, while this was not the case among saproxylic species. The mechanisms by which non-saproxylic species may have benefitted from the outbreak are unclear. With respect to *O. laqueatus*, this species has been recorded in many types of decaying organic matter and also from sap runs [62]. Thus, we cannot rule out that this species has somehow benefitted from increased availability of dead wood, even though its substrate preferences seem to be too wide to justify classifying it as saproxylic. The finding of clear community changes in non-saproxylics means that our inferences about numerical responses to the outbreak, which were partly based on contrasting the three DWA groups, must be treated with some caution. Clearly, non-saproxylics cannot be viewed as an unaffected control group with respect to the outbreak. Indeed, the pervasive impact of the outbreak on the whole ecosystem, including understory vegetation, herbivores [14], [63] and abiotic conditions [64], probably leaves few biological taxa unaffected. However, this does not change our main conclusion about weak numerical responses in the saproxylic beetle community.

Although the RDA indicates that beetle community structure was affected by the outbreak, it should be emphasised that forest damage explained a small fraction of the overall variance in the beetle data (at most 11.1%, in the case of obligate saproxylics). Thus, although the outbreak represents a dramatic disturbance, which has changed both the biotic and abiotic environment, its effect on many beetle species may be overruled by other environmental variables. This conclusion is supported by the fact that counts of the DWA groups and many beetle species showed large-scale spatial gradients that appeared to be independent of forest damage. Notably though, these gradients may to some extent also represent variation in trapping efficiency or beetle activity, due to variation in e.g. wind speed or sun-exposure along the transects. Both transects have considerable heterogeneity in topographical variables, such as altitude and aspect (Fig. S2 in Appendix S1), and this may have affected both trapping efficiencies and beetle activity patterns.

## Conclusions

Three to five years after an extensive moth outbreak, the saproxylic beetle community in the sub-arctic mountain birch forest appears to have mounted a weak but detectable numerical response to the increased availability of dead wood in the system. This response has mainly been driven by a few very abundant species, which utilize dead wood in early stages of decay. Although further monitoring is required to assess the full extent of beetle responses to the outbreak, our present results would suggest the possibility that saproxylic beetles increase in abundance to slowly to play a major role in decomposing the enormous quantities of dead wood left by moth outbreaks in this system. Accordingly, the role of microbial decomposer communities in post-outbreak decomposition dynamics represents a prime topic for future research.

## Supporting Information

Appendix S1 Supporting tables and figures. Table S1 List of all beetle species encountered in the study, with information on association with dead wood birch. Table S2 Results of model selection for log-linear models for DWA groups. Table S3 Coefficients from selected log-linear and logistic models for DWA groups. Table S4 Results of model selection for logistic models for DWA groups. Table S5 Results of model selection for log-linear models for individual beetle species. Table S6 Coefficients from selected log-linear models for individual beetle species. Figure S1 Photographs from the individual sampling stations in Kirkenes and Tana in early June 2011. Figure S2 Station-specific scores for topographic variables.
(DOCX)

## References

[1] Barbosa P, Letourneau D, Agrawal A (2012) Insect Outbreaks Revisited: Wiley.

[2] Yang LH (2012) The Ecological Consequences of Insect Outbreaks. Insect Outbreaks Revisited: John Wiley & Sons, Ltd. pp. 197–218.

[3] Haney JC (1999) Numerical response of birds to an irruption of elm spanworm (Ennomos subsignarius [Hbn.]; Geometridae: Lepidoptera) in old-growth forest of the Appalachian Plateau, USA. Forest Ecology and Management 120: 203–217.

[4] Drever MC, Goheen JR, Martin K (2009) Species-Energy Theory, Pulsed Resources, and Regulation of Avian Richness during a Mountain Pine Beetle Outbreak. Ecology 90: 1095–1105. 19449703 10.1890/08-0575.1

[5] Hogstad O (2005) Numerical and functional responses of breeding passerine species to mass occurrence of geometrid caterpillars in a subalpine birch forest: a 30-year study. Ibis 147: 77–91.

[6] Marcello GJ, Wilder SM, Meikle DB (2008) Population dynamics of a generalist rodent in relation to variability in pulsed food resources in a fragmented landscape. Journal of Animal Ecology 77: 41–46. 17976166 10.1111/j.1365-2656.2007.01310.x

[7] Vandegrift KJ, Hudson PJ (2009) Response to enrichment, type and timing: small mammals vary in their response to a springtime cicada but not a carbohydrate pulse. Journal of Animal Ecology 78: 202–209. 18684131 10.1111/j.1365-2656.2008.01456.x

[8] Reeve JD (1997) Predation and bark beetle dynamics. Oecologia 112: 48–54. 28307375 10.1007/s004420050282

[9] Eveleigh ES, McCann KS, McCarthy PC, Pollock SJ, Lucarotti CJ, et al (2007) Fluctuations in density of an outbreak species drive diversity cascades in food webs. Proceedings of the National Academy of Sciences 104: 16976–16981. 10.1073/pnas.0704301104 PMC2040472 17940003

[10] Yang LH (2004) Periodical Cicadas as Resource Pulses in North American Forests. Science 306: 1565–1567. 15567865 10.1126/science.1103114

[11] Eschtruth AK, Cleavitt NL, Battles JJ, Evans RA, Fahey TJ (2006) Vegetation dynamics in declining eastern hemlock stands: 9 years of forest response to hemlock woolly adelgid infestation. Canadian Journal of Forest Research 36: 1435–1450.

[12] Gandhi KK, Herms D (2010) Direct and indirect effects of alien insect herbivores on ecological processes and interactions in forests of eastern North America. Biological Invasions 12: 389–405.

[13] Bell JL, Whitmore RC (1997) Bird Populations and Habitat in Bacillus thuringiensis and Dimilin-treated and Untreated Areas of Hardwood Forest. American Midland Naturalist 137: 239–250.

[14] Jepsen J, Biuw M, Ims R, Kapari L, Schott T, et al (2013) Ecosystem Impacts of a Range Expanding Forest Defoliator at the Forest-Tundra Ecotone. Ecosystems 16: 561–575.

[15] Yang LH, Bastow JL, Spence KO, Wright AN (2008) What can we learn from resource pulses? Ecology 89: 621–634. 18459327 10.1890/07-0175.1

[16] Yang LH, Edwards KF, Byrnes JE, Bastow JL, Wright AN, et al (2010) A meta-analysis of resource pulse-consumer interactions. Ecological Monographs 80: 125–151.

[17] Speight MCD (1989) Saproxylic invertebrates and their conservation: Council of Europe.

[18] Muona J, Rutanen I (1994) The short-term impact of tire on the beetle fauna in bored coniferous forest. Annales Zoologici Fennici 31: 109–121.

[19] Saint-Germain M, Drapeau P, Hébert C (2004) Landscape-Scale Habitat Selection Patterns of Monochamus scutellatus (Coleoptera: Cerambycidae) in a Recently Burned Black Spruce Forest. Environmental Entomology 33: 1703–1710.

[20] Boulanger Y, Sirois L (2007) Postfire Succession of Saproxylic Arthropods, with Emphasis on Coleoptera, in the North Boreal Forest of Quebec. Environmental Entomology 36: 128–141. 17349126 10.1603/0046-225X-36.1.128

[21] Wermelinger B, Duelli P, Obrist MK (2002) Dynamics of saproxylic beetles (Coleoptera) in windthrow areas in alpine spruce forests. Forest snow and landscape research 77: 133–148.

[22] Bouget C, Duelli P (2004) The effects of windthrow on forest insect communities: a literature review. Biological Conservation 118: 281–299.

[23] Grimbacher P, Stork N (2009) How do beetle assemblages respond to cyclonic disturbance of a fragmented tropical rainforest landscape? Oecologia 161: 591–599. 19597849 10.1007/s00442-009-1399-5

[24] Müller J, Noss RF, Bussler H, Brandl R (2010) Learning from a “benign neglect strategy” in a national park: Response of saproxylic beetles to dead wood accumulation. Biological Conservation 143: 2559–2569.

[25] Ehnström B, Axelsson R (2002) Insektsgnag i bark och ved: ArtDatabanken SLU.

[26] Langor DW, Spence JR, Hammond HJ (2004) Saproxylic beetles (Coleoptera) using Populus in boreal aspen stands of western Canada: spatiotemporal variation and conservation of assemblages. Canadian Journal of Forest Research 34: 1–19.

[27] Jogeir N. Stokland JS, Jonsson BG (2012) Biodiversity in Dead Wood: Cambridge University Press.

[28] Price PW, Denno RF, Eubanks MD, Finke DL, Kaplan I (2011) Insect Ecology: Behavior, Populations and Communities: Cambridge University Press.

[29] Schowalter TD, Caldwell BC, Carpenter SE, Griffiths RP, Harmon ME, et al.. (1992) Decomposition of fallen trees: effects of initial conditions and heterotroph colonization rates. In: Singh KP, Singh JS, editors. Tropical Ecosystems: Ecology and Management. New Delhi: Wiley Eastern Limited. pp. 373–383.

[30] Zhong H, Schowalter TD (1989) Conifer bole utilization by wood-boring beetles in western Oregon. Canadian Journal of Forest Research 19: 943–947.

[31] Muller MM, Varama M, Heinonen J, Hallaksela AM (2002) Influence of insects on the diversity of fungi in decaying spruce wood in managed and natural forests. Forest Ecology and Management 166: 165–181.

[32] Müller J, Buβler H, Goβner M, Rettelbach T, Duelli P (2008) The European spruce bark beetle Ips typographus in a national park: from pest to keystone species. Biodiversity and Conservation 17: 2979–3001.

[33] Lehnert LW, Bässler C, Brandl R, Burton PJ, Müller J (2013) Conservation value of forests attacked by bark beetles: Highest number of indicator species is found in early successional stages. Journal for Nature Conservation 21: 97–104.

[34] Jepsen JU, Hagen SB, Karlsen SR, Ims RA (2009) Phase-dependent outbreak dynamics of geometrid moth linked to host plant phenology. Proceedings of the Royal Society B-Biological Sciences 276: 4119–4128. 10.1098/rspb.2009.1148 PMC2821342 19740876

[35] Kålås JA, Viken Å, Henriksen S, Skjelseth S (2010) The 2010 Norwegian Red List for Species. Norway: Norwegian Biodiversity Information Centre.

[36] Tenow O (1972) The outbreaks of Oporinia autumnata Bkh. and Operophtera spp. (Lep., Geometridae) in the Scandinavian mountain chain and northern Finland 1862–1968. Zoologiska bidrag från Uppsala, Supplement, 2: , 1–107.

[37] Bylund H (1999) Climate and the population dynamics of two insect outbreak species in the north. Ecological Bulletins 47: 54–62.

[38] Ruohomäki K, Tanhuanpää M, Ayres MP, Kaitaniemi P, Tammaru T, et al (2000) Causes of cyclicity of Epirrita autumnata (Lepidoptera, Geometridae): grandiose theory and tedious practice. Population Ecology 42: 211–223.

[39] Kaila L, Martikainen P, Punttila P (1997) Dead trees left in clear-cuts benefit saproxylic Coleoptera adapted to natural disturbances in boreal forest. Biodiversity & Conservation 6: 1–18.

[40] Dahlberg A, Stokland JN (2004) Vedlevande arters krav på substrat: sammanställning och analys av 3 600 arter: Skogsstyrelsen.

[41] Böhme J (2005) Die Käfer Mitteleuropas: Katalog. München: Elsevier Spektrum Akademischer Verlag.

[42] Legendre P, Gallagher ED (2001) Ecologically Meaningful Transformations for Ordination of Species Data. Oecologia 129: 271–280. 28547606 10.1007/s004420100716

[43] Ulyshen MD (2013) Strengthening the case for saproxylic arthropod conservation: a call for ecosystem services research. Insect Conservation and Diversity 6: 393–395.

[44] Ulyshen MD, Wagner TL (2013) Quantifying arthropod contributions to wood decay. Methods in Ecology and Evolution 4: 345–352.

[45] Gullan PJ, Cranston PS (2010) The Insects: An Outline of Entomology: Wiley.

[46] Martikainen P, Kaila L (2004) Sampling saproxylic beetles: lessons from a 10-year monitoring study. Biological Conservation 120: 171–181.

[47] Menke N (2006) Untersuchungen zur Struktur und Sukzession der saproxylen Käferfauna (Coleoptera) an Eichen- und Buchentotholz: Georg-August-Universität Göttingen.

[48] Sverdrup-Thygeson A, Birkemoe T (2009) What window traps can tell us: effect of placement, forest openness and beetle reproduction in retention trees. Journal of Insect Conservation 13: 183–191.

[49] Brown JH, Mehlman DW, Stevens GC (1995) Spatial Variation in Abundance. Ecology 76: 2028–2043.

[50] Jonsell M, Weslien J, Ehnström B (1998) Substrate requirements of red-listed saproxylic invertebrates in Sweden. Biodiversity & Conservation 7: 749–764.

[51] Lindhe A, Lindelöw Å, Åsenblad N (2005) Saproxylic Beetles in Standing Dead Wood Density in Relation to Substrate Sun-exposure and Diameter. Biodiversity & Conservation 14: 3033–3053.

[52] Sverdrup-Thygeson A, Ims RA (2002) The effect of forest clearcutting in Norway on the community of saproxylic beetles on aspen. Biological Conservation 106: 347–357.

[53] Gibb H, Hjältén J, P. Ball J, Atlegrim O, Pettersson RB, et al (2006) Effects of landscape composition and substrate availability on saproxylic beetles in boreal forests: a study using experimental logs for monitoring assemblages. Ecography 29: 191–204.

[54] Bergman K-O, Jansson N, Claesson K, Palmer MW, Milberg P (2012) How much and at what scale? Multiscale analyses as decision support for conservation of saproxylic oak beetles. Forest Ecology and Management 265: 133–141.

[55] Sverdrup-Thygeson A, Gustafsson L, Kouki J (2014) Spatial and temporal scales relevant for conservation of dead-wood associated species: current status and perspectives. Biodiversity and Conservation 23: 513–535.

[56] Mäkinen H, Hynynen J, Siitonen J, Sievänen R (2006) Predicting the Decomposition of Scots Pine, Norway Spruce, and Birch Stems in Finland. Ecological Applications 16: 1865–1879. 17069378 10.1890/1051-0761(2006)016[1865:ptdosp]2.0.co;2

[57] Martikainen P, Siitonen J, Punttila P, Kaila L, Rauh J (2000) Species richness of Coleoptera in mature managed and old-growth boreal forests in southern Finland. Biological Conservation 94: 199–209.

[58] Jonsson B, Kruys N, Ranius T (2005) Ecology of species living on dead wood-Lessons for dead wood management. Silva Fennica 39: 289–309.

[59] Hyvärinen E (2006) Green-tree retention and controlled burning in restoration and conservation of beetle diversity in boreal forests [Ph. D]: University of Joensuu.

[60] Gustafsson L, Kouki J, Sverdrup-Thygeson A (2010) Tree retention as a conservation measure in clear-cut forests of northern Europe: a review of ecological consequences. Scandinavian Journal of Forest Research 25: 295–308.

[61] Lindenmayer DB, Franklin JF, Lõhmus A, Baker SC, Bauhus J, et al (2012) A major shift to the retention approach for forestry can help resolve some global forest sustainability issues. Conservation Letters 5: 421–431.

[62] Buckland PI, Buckland PC (2006) BugsCEP Coleopteran Ecology Package. IGBP PAGES/World Data Center for Paleoclimatology Data Contribution Series # 2006-116. NOAA/NCDC Paleoclimatology Program, Boulder CO, USA. URL: http://www.ncdc.noaa.gov/paleo/insect.html.

[63] Karlsen S, Jepsen J, Odland A, Ims R, Elvebakk A (2013) Outbreaks by canopy-feeding geometrid moth cause state-dependent shifts in understorey plant communities. Oecologia: 1–12. 10.1007/s00442-013-2648-1 PMC3824357 23568711

[64] Kaukonen M, Ruotsalainen AL, Wäli PR, Männistö MK, Setälä H, et al (2012) Moth herbivory enhances resource turnover in subarctic mountain birch forests? Ecology 94: 267–272. 10.1890/12-0917.1 23691644

[65] Greenacre MJ (2010) Biplots in Practice: Fundación BBVA.

[66] Wilkinson L (2012) Exact and Approximate Area-Proportional Circular Venn and Euler Diagrams. IEEE Transactions on Visualization and Computer Graphics 18: 321–331. 21383412 10.1109/TVCG.2011.56

