# Supplementary material for: Numerical Responses of Saproxylic Beetles to Rapid Increases in Dead Wood Availability following Geometrid Moth Outbreaks in Sub-Arctic Mountain Birch Forest
Source: PLoS One. 2014 Jun 9;9(6):e99624. doi: 10.1371/journal.pone.0099624 (PMC4049814; doi:10.1371/journal.pone.0099624)
Supplement: Appendix S1 — Supporting tables and figures. Table S1 List of all beetle species encountered in the study, with information on association with dead wood birch. Table S2 Results of model selection for log-linear models for DWA groups. Table S3 Coefficients from selected log-linear and logistic models for DWA groups. Table S4 Results of model selection for logistic models for DWA groups. Table S5 Results of model selection for log-linear models for individual beetle species. Table S6 Coefficients from selected log-linear models for individual beetle species. Figure S1 Photographs from the individual sampling stations in Kirkenes and Tana in early June 2011. Figure S2 Station-specific scores for topographic variables. (DOCX) [file pone.0099624.s001.docx]

**Appendix S1. Supporting tables and figures**

**Table S1**. List of all beetle species encountered in the study, with information on association with dead wood [obligate saproxylic (a), facultative saproxylic (b), non-saproxylic (c) and unknown dead wood association (d)], larval trophic guild (P = Predator, F = Fungivore, H = Herbivore, W = Wood-feeding, S = Several, O = Other and NA = Unknown) and association with birch (Y = Birch associated, N = Not birch associated and NA = Unknown). For species with several trophic guilds, the relevant guilds are given in parenthesis. The table also shows the sources that have been used to classify the species (reference list at the bottom of the table), and the total individual count for each species across both study years in the dead (D) and living (L) sections of the Kirkenes and Tana transects.

| a) Obligate saproxylic |  |  |  | Kirkenes | |  | Tana | |  |
| --- | --- | --- | --- | --- | --- | --- | --- | --- | --- |
| Species | Family | Guild | Birch | D | L |  | D | L | Ref. |
| *Anisotoma castanea* | Leiodidae | F | Y | 2 | 0 |  | 0 | 0 | 1, 2 |
| *Atheta diversa* | Staphylinidae | P | NA | 7 | 0 |  | 3 | 0 | 1, 4 |
| *Atheta taxiceroides* | Staphylinidae | F | Y | 3 | 1 |  | 2 | 0 | 1, 3 |
| *Atrecus pilicornis* | Staphylinidae | P | Y | 0 | 0 |  | 1 | 0 | 1, 2 |
| *Dadobia immersa* | Staphylinidae | S (P, F) | Y | 3 | 6 |  | 1 | 2 | 1, 2, 4 |
| *Dinaraea aequata* | Staphylinidae | S (P, F) | Y | 0 | 1 |  | 0 | 0 | 1, 2, 4 |
| *Eudectus giraudi* | Staphylinidae | F | Y | 6 | 14 |  | 21 | 13 | 2, 5, 6 |
| *Euplectus punctatus* | Staphylinidae | P | Y | 6 | 21 |  | 5 | 10 | 1, 4 |
| *Leptusa pulchella* | Staphylinidae | S (P, F) | Y | 0 | 1 |  | 0 | 0 | 1, 2, 4 |
| *Lordithon speciosus* | Staphylinidae | P | Y | 0 | 1 |  | 0 | 0 | 1, 2 |
| *Olisthaerus megacephalus* | Staphylinidae | F | Y | 0 | 1 |  | 1 | 0 | 1, 2 |
| *Phloeopora corticalis* | Staphylinidae | P | Y | 4 | 6 |  | 5 | 0 | 1, 2 |
| *Placusa tachyporoides* | Staphylinidae | P | Y | 0 | 1 |  | 0 | 0 | 1, 2 |
| *Ampedus nigrinus* | Elateridae | S (P, W) | Y | 1 | 1 |  | 0 | 0 | 2, 4 |
| *Denticollis borealis* | Elateridae | S (P, W) | Y | 0 | 0 |  | 1 | 0 | 2, 3 |
| *Denticollis linearis* | Elateridae | S (P, W) | Y | 9 | 18 |  | 20 | 23 | 2, 4 |
| *Diacanthous undulatus* | Elateridae | NA | Y | 0 | 0 |  | 1 | 3 | 5 |
| *Sericus brunneus* | Elateridae | W | N | 16 | 5 |  | 20 | 11 | 1, 2 |
| *Triplax aenea* | Erotylidae | F | Y | 2 | 0 |  | 0 | 1 | 1, 2 |
| *Triplax scutellaris* | Erotylidae | F | Y | 7 | 1 |  | 2 | 1 | 1, 2 |
| *Malthodes brevicollis* | Cantharidae | P | Y | 2 | 8 |  | 2 | 5 | 1, 2 |
| *Malthodes fuscus* | Cantharidae | P | NA | 2 | 2 |  | 0 | 1 | 1, 2 |
| *Malthodes guttifer* | Cantharidae | P | N | 57 | 26 |  | 11 | 63 | 1, 2 |
| *Podistra schoenherri* | Cantharidae | P | Y | 22 | 27 |  | 32 | 46 | 1, 2 |
| *Elateroides dermestoides* | Lymexylidae | F | Y | 436 | 26 |  | 123 | 28 | 1 |
| *Aplocnemus tarsalis* | Melyridae | P | N | 3 | 0 |  | 0 | 0 | 1, 2 |
| *Dasytes obscurus* | Melyridae | P | N | 1 | 0 |  | 0 | 0 | 1, 2 |
| *Epuraea angustula* | Nitidulidae | S (P, F) | Y | 2 | 1 |  | 0 | 0 | 1, 2, 7 |
| *Epuraea boreella* | Nitidulidae | S (P, F) | Y | 1 | 0 |  | 0 | 1 | 1, 2, 7 |
| *Glischrochilus quadripunctatus* | Nitidulidae | S (P, F) | Y | 0 | 0 |  | 1 | 0 | 1, 2, 6 |
| *Pediacus fuscus* | Silvanidae | P | Y | 1 | 0 |  | 0 | 0 | 1, 2 |
| Obligate saproxylic (continued) |  |  |  | Kirkenes | |  | Tana | |  |
| Species | Family | Guild | Birch | D | L |  | D | L | Ref. |
| *Cryptophagus tuberculosus* | Cryptophagidae | F | NA | 0 | 0 |  | 0 | 1 | 8 |
| *Cerylon ferrugineum* | Cerylonidae | S (P, F) | Y | 2 | 36 |  | 11 | 5 | 1, 2 |
| *Corticaria orbicollis* | Latridiidae | F | Y | 0 | 5 |  | 5 | 2 | 1, 2 |
| *Enicmus fungicola* | Latridiidae | F | Y | 14 | 34 |  | 4 | 3 | 1, 2 |
| *Enicmus lundbladi* | Latridiidae | F | N | 0 | 1 |  | 2 | 0 | 2, 3 |
| *Cis bidentatus* | Ciidae | F | Y | 1 | 0 |  | 0 | 0 | 1, 2 |
| *Cis boleti* | Ciidae | F | Y | 5 | 2 |  | 4 | 1 | 1, 2 |
| *Cis comptus* | Ciidae | F | Y | 0 | 0 |  | 2 | 0 | 1, 2 |
| *Cis micans* | Ciidae | F | Y | 0 | 1 |  | 0 | 0 | 1, 2 |
| *Orthocis alni* | Ciidae | F | Y | 3 | 5 |  | 3 | 6 | 1, 2 |
| *Tetratoma ancora* | Tetratomidae | F | Y | 2 | 5 |  | 2 | 0 | 1, 2 |
| *Orchesia micans* | Melandryidae | F | Y | 1 | 2 |  | 2 | 1 | 1, 2 |
| *Orchesia minor* | Melandryidae | F | Y | 2 | 0 |  | 0 | 0 | 1, 2 |
| *Rabocerus foveolatus* | Salpingidae | P | Y | 66 | 43 |  | 40 | 5 | 1, 2 |
| *Salpingus ruficollis* | Salpingidae | P | Y | 39 | 18 |  | 5 | 0 | 1, 2 |
| *Anaspis arctica* | Scraptiidae | S (P, F, W) | Y | 2 | 3 |  | 1 | 14 | 1, 2, 9 |
| *Rhagium mordax* | Cerambycidae | W | Y | 10 | 1 |  | 0 | 0 | 1, 2 |
| *Hylastes brunneus* | Curculionidae | W | N | 0 | 0 |  | 1 | 0 | 1, 2 |
| *Pityogenes bidentatus* | Curculionidae | W | N | 1 | 0 |  | 2 | 1 | 1, 2 |
| *Pityogenes chalcographus* | Curculionidae | W | N | 0 | 1 |  | 1 | 1 | 1, 2 |
| *Trypodendron signatum* | Curculionidae | F | Y | 8 | 0 |  | 0 | 0 | 1, 2 |
| b) Facultative saproxylic |  |  |  |  |  |  |  |  |  |
| *Dromius agilis* | Carabidae | P | Y | 0 | 0 |  | 1 | 2 | 1, 2 |
| *Sphaerites glabratus* | Sphaeritidae | NA | NA | 1 | 0 |  | 0 | 0 | 10, 11 |
| *Acrotrichis rugulosa* | Ptiliidae | F | Y | 0 | 0 |  | 1 | 0 | 1, 2 |
| *Agathidium arcticum* | Leiodidae | F | Y | 0 | 0 |  | 2 | 0 | 1, 2 |
| *Agathidium confusum* | Leiodidae | F | Y | 0 | 1 |  | 0 | 0 | 1, 2 |
| *Agathidium rotundatum* | Leiodidae | F | Y | 2 | 1 |  | 0 | 1 | 1, 2 |
| *Stenichnus bicolor* | Scydmaenidae | P | Y | 1 | 1 |  | 2 | 4 | 2, 4 |
| *Acidota crenata* | Staphylinidae | P | NA | 4 | 3 |  | 8 | 1 | 1, 2 |
| *Acrulia inflata* | Staphylinidae | S (P, F) | Y | 0 | 0 |  | 1 | 0 | 1, 2 |
| *Arpedium quadrum* | Staphylinidae | P | NA | 0 | 0 |  | 1 | 0 | 2, 12 |
| *Atheta aeneipennis* | Staphylinidae | P | NA | 2 | 0 |  | 2 | 0 | 1, 2 |
| *Atheta allocera* | Staphylinidae | P | NA | 8 | 4 |  | 3 | 1 | 2, 12 |
| *Atheta atramentaria* | Staphylinidae | NA | NA | 0 | 0 |  | 2 | 0 | 12 |
| *Atheta hypnorum* | Staphylinidae | P | NA | 20 | 14 |  | 21 | 22 | 1, 2 |
| *Atheta laticollis* | Staphylinidae | P | NA | 0 | 0 |  | 3 | 0 | 11 |
| *Atheta myrmecobia* | Staphylinidae | P | NA | 2 | 0 |  | 1 | 0 | 2, 12 |
| *Atheta pilicornis* | Staphylinidae | S (P, F) | NA | 0 | 0 |  | 1 | 0 | 1, 2, 4 |
| *Atheta procera* | Staphylinidae | P | NA | 3 | 1 |  | 1 | 0 | 2, 12 |
| *Bolitochara pulchra* | Staphylinidae | P | Y | 0 | 1 |  | 0 | 0 | 1, 2 |
| Facultative saproxylic (continued) |  |  |  | Kirkenes | |  | Tana | |  |
| Species | Family | Guild | Birch | D | L |  | D | L | Ref. |
| *Bryophacis rufus punctipennis* | Staphylinidae | P | NA | 1 | 0 |  | 0 | 1 | 2, 12 |
| *Bryoporus cernuus* | Staphylinidae | P | NA | 0 | 2 |  | 3 | 0 | 2, 12 |
| *Coryphium angusticolle* | Staphylinidae | P | Y | 0 | 0 |  | 1 | 0 | 1, 4 |
| *Euplectus karstenii* | Staphylinidae | P | Y | 0 | 4 |  | 1 | 0 | 2, 4 |
| *Euplectus signatus* | Staphylinidae | P | NA | 0 | 2 |  | 0 | 7 | 2, 9 |
| *Gabrius appendiculatus* | Staphylinidae | P | NA | 0 | 1 |  | 0 | 0 | 2, 11 |
| *Ischnoglossa prolixa* | Staphylinidae | P | Y | 0 | 1 |  | 1 | 0 | 1, 2 |
| *Lordithon thoracicus* | Staphylinidae | P | Y | 0 | 0 |  | 1 | 0 | 1, 2 |
| *Megarthrus prosseni* | Staphylinidae | P | NA | 2 | 0 |  | 1 | 1 | 1, 2 |
| *Mniusa incrassata* | Staphylinidae | P | Y | 0 | 0 |  | 1 | 1 | 1, 2 |
| *Mycetoporus maerkeli* | Staphylinidae | F | NA | 0 | 0 |  | 1 | 0 | 2, 13 |
| *Mycetoporus punctus* | Staphylinidae | F | NA | 4 | 2 |  | 6 | 0 | 1, 2 |
| *Omalium strigicolle* | Staphylinidae | P | NA | 6 | 0 |  | 4 | 3 | 2, 14 |
| *Quedius plagiatus* | Staphylinidae | P | Y | 8 | 11 |  | 1 | 1 | 1, 2 |
| *Scaphisoma agaricinum* | Staphylinidae | F | Y | 1 | 0 |  | 0 | 0 | 1, 2 |
| *Tachinus lignorum* | Staphylinidae | P | NA | 0 | 0 |  | 1 | 0 | 2, 11 |
| *Liotrichus affinis* | Elateridae | NA | Y | 114 | 89 |  | 59 | 157 | 12 |
| *Paraphotistus impressus* | Elateridae | P | NA | 2 | 3 |  | 0 | 6 | 12 |
| *Aspidiphorus orbiculatus* | Sphindidae | F | Y | 0 | 2 |  | 0 | 0 | 2, 4 |
| *Epuraea aestiva* | Nitidulidae | S (P, F) | NA | 4 | 16 |  | 1 | 6 | 1, 2, 7 |
| *Epuraea melina* | Nitidulidae | S (P, F) | N | 0 | 0 |  | 0 | 1 | 2, 4, 7 |
| *Epuraea rufomarginata* | Nitidulidae | S (P, F) | Y | 1 | 2 |  | 0 | 0 | 1, 2, 7 |
| *Cryptophagus lapponicus* | Cryptophagidae | F | Y | 2 | 6 |  | 4 | 4 | 1, 2 |
| *Corticaria ferruginea* | Latridiidae | F | Y | 0 | 7 |  | 1 | 2 | 1, 2 |
| *Corticaria rubripes* | Latridiidae | F | Y | 2 | 0 |  | 1 | 1 | 1, 2 |
| *Dienerella filum* | Latridiidae | F | N | 0 | 0 |  | 0 | 1 | 1, 2 |
| *Latridius consimilis* | Latridiidae | F | Y | 0 | 2 |  | 1 | 2 | 1, 2 |
| *Latridius minutus* | Latridiidae | F | Y | 0 | 1 |  | 0 | 2 | 1, 2 |
| c) Non-saproxylic |  |  |  |  |  |  |  |  |  |
| *Agabus congener* | Dytiscidae | P | - | 0 | 1 |  | 0 | 0 | 2 |
| *Agonum consimile* | Carabidae | P | - | 0 | 1 |  | 0 | 0 | 15 |
| *Amara apricaria* | Carabidae | H | - | 0 | 0 |  | 1 | 0 | 15 |
| *Bembidion grapii* | Carabidae | P | - | 0 | 1 |  | 0 | 0 | 15 |
| *Cercyon lateralis* | Hydrophilidae | O | - | 1 | 0 |  | 2 | 0 | 2 |
| *Cercyon melanocephalus* | Hydrophilidae | O | - | 1 | 1 |  | 4 | 0 | 2 |
| *Cryptopleurum minutum* | Hydrophilidae | O | - | 0 | 0 |  | 2 | 0 | 2 |
| *Megasternum concinnum* | Hydrophilidae | O | - | 3 | 1 |  | 0 | 0 | 2 |
| *Acrotrichis cognata* | Ptiliidae | F | - | 6 | 1 |  | 0 | 0 | 2, 16 |
| *Acrotrichis intermedia* | Ptiliidae | F | - | 5 | 0 |  | 1 | 0 | 2, 16 |
| *Acrotrichis parva* | Ptiliidae | F | - | 0 | 0 |  | 2 | 0 | 2, 16 |
| *Acrotrichis sericans* | Ptiliidae | F | - | 9 | 4 |  | 6 | 2 | 2, 11 |
| Non-saproxylic (continued) |  |  |  | Kirkenes | |  | Tana | |  |
| Species | Family | Guild | Birch | D | L |  | D | L | Ref. |
| *Acrotrichis strandi* | Ptiliidae | F | - | 0 | 2 |  | 0 | 0 | 2, 11 |
| *Catops alpinus* | Leiodidae | NA | - | 3 | 1 |  | 0 | 2 | 14 |
| *Catops tristis* | Leiodidae | NA | - | 1 | 0 |  | 0 | 0 | 2 |
| *Colon serripes* | Leiodidae | F | - | 0 | 0 |  | 1 | 0 | 2 |
| *Hydnobius septentrionalis* | Leiodidae | F | - | 0 | 0 |  | 1 | 0 | 17 |
| *Leiodes inordinata* | Leiodidae | F | - | 2 | 0 |  | 0 | 0 | 17 |
| *Leiodes obesa* | Leiodidae | F | - | 1 | 0 |  | 0 | 0 | 2 |
| *Nicrophorus vespilloides* | Silphidae | O | - | 1 | 0 |  | 3 | 0 | 2 |
| *Acrostiba borealis* | Staphylinidae | NA | - | 23 | 18 |  | 12 | 4 | 14 |
| *Acrotona fungi* | Staphylinidae | P | - | 8 | 8 |  | 43 | 9 | 2 |
| *Acrotona orbata* | Staphylinidae | P | - | 0 | 0 |  | 2 | 0 | 2 |
| *Aleochara brundini* | Staphylinidae | P | - | 0 | 0 |  | 1 | 0 | 2 |
| *Amischa analis* | Staphylinidae | P | - | 1 | 0 |  | 0 | 0 | 2, 18 |
| *Anthophagus alpinus* | Staphylinidae | P | - | 32 | 2 |  | 11 | 22 | 2 |
| *Anthophagus omalinus* | Staphylinidae | P | - | 663 | 411 |  | 96 | 228 | 2 |
| *Atheta cinnamoptera* | Staphylinidae | P | - | 6 | 0 |  | 6 | 1 | 2 |
| *Atheta debilis* | Staphylinidae | P | - | 2 | 0 |  | 2 | 0 | 2 |
| *Atheta depressicollis* | Staphylinidae | P | - | 1 | 3 |  | 0 | 0 | 2, 14 |
| *Atheta elongatula* | Staphylinidae | P | - | 0 | 0 |  | 1 | 0 | 2 |
| *Atheta excellens* | Staphylinidae | P | - | 3 | 0 |  | 2 | 0 | 2, 11 |
| *Atheta graminicola* | Staphylinidae | P | - | 3 | 2 |  | 18 | 7 | 14 |
| *Atheta melanocera* | Staphylinidae | P | - | 0 | 0 |  | 7 | 2 | 2, 11 |
| *Atheta palleola* | Staphylinidae | P | - | 1 | 0 |  | 0 | 0 | 14 |
| *Atheta setigera* | Staphylinidae | P | - | 0 | 0 |  | 1 | 0 | 2, 11 |
| *Atheta strandiella* | Staphylinidae | P | - | 0 | 0 |  | 1 | 0 | 2, 11 |
| *Autalia puncticollis* | Staphylinidae | P | - | 0 | 0 |  | 2 | 0 | 2, 14 |
| *Bisnius nigriventris* | Staphylinidae | P | - | 0 | 0 |  | 1 | 0 | 2 |
| *Bryophacis maklini* | Staphylinidae | P | - | 7 | 3 |  | 16 | 2 | 2 |
| *Eucnecosum brachypterum* | Staphylinidae | P | - | 0 | 0 |  | 1 | 0 | 2 |
| *Eusphalerum lapponicum* | Staphylinidae | H | - | 0 | 1 |  | 0 | 0 | 2 |
| *Lordithon trinotatus* | Staphylinidae | S (P, F) | - | 1 | 0 |  | 0 | 0 | 2, 11 |
| *Lypoglossa lateralis* | Staphylinidae | P | - | 1 | 1 |  | 3 | 1 | 2 |
| *Megarthrus nigrinus* | Staphylinidae | P | - | 2 | 0 |  | 2 | 0 | 19 |
| *Megarthrus nitidulus* | Staphylinidae | P | - | 0 | 1 |  | 0 | 0 | 2 |
| *Mycetoporus lepidus* | Staphylinidae | F | - | 3 | 2 |  | 1 | 0 | 2, 24 |
| *Mycetoporus mulsanti* | Staphylinidae | F | - | 4 | 0 |  | 1 | 0 | 2, 14 |
| *Notothecta flavipes* | Staphylinidae | P | - | 0 | 1 |  | 1 | 1 | 2, 14 |
| *Olophrum consimile* | Staphylinidae | P | - | 2 | 1 |  | 1 | 1 | 2, 11 |
| *Omalium septentrionis* | Staphylinidae | P | - | 0 | 0 |  | 1 | 0 | 2, 11 |
| *Oxypoda brevicornis* | Staphylinidae | P | - | 1 | 2 |  | 10 | 3 | 24 |
| *Oxypoda haemorrhoa* | Staphylinidae | P | - | 0 | 1 |  | 0 | 0 | 2 |
| *Oxypoda nigricornis* | Staphylinidae | P | - | 0 | 0 |  | 1 | 0 | 2, 14 |
| Non-saproxylic (continued) |  |  |  | Kirkenes | |  | Tana | |  |
| Species | Family | Guild | Birch | D | L |  | D | L | Ref. |
| *Oxypoda skalitzkyi* | Staphylinidae | P | - | 2 | 2 |  | 4 | 0 | 2, 14 |
| *Oxytelus laqueatus* | Staphylinidae | P | - | 77 | 15 |  | 54 | 5 | 4, 24 |
| *Philonthus albipes* | Staphylinidae | NA | - | 0 | 0 |  | 2 | 0 | 11 |
| *Philonthus corvinus* | Staphylinidae | P | - | 1 | 0 |  | 0 | 0 | 2 |
| *Stenus geniculatus* | Staphylinidae | P | - | 0 | 0 |  | 3 | 0 | 11 |
| *Tachinus elongatus* | Staphylinidae | NA | - | 14 | 2 |  | 10 | 1 | 2 |
| *Tachinus laticollis* | Staphylinidae | NA | - | 2 | 0 |  | 8 | 0 | 2 |
| *Tachinus pallipes* | Staphylinidae | P | - | 3 | 0 |  | 1 | 0 | 2 |
| *Tachyporus chrysomelinus* | Staphylinidae | P | - | 0 | 0 |  | 0 | 1 | 2 |
| *Tetartopeus zetterstedti* | Staphylinidae | P | - | 1 | 0 |  | 0 | 0 | 2, 14 |
| *Aphodius fasciatus* | Scarabaeidae | O | - | 0 | 0 |  | 4 | 0 | 2 |
| *Aphodius lapponum* | Scarabaeidae | O | - | 19 | 4 |  | 5 | 0 | 2 |
| *Aphodius piceus* | Scarabaeidae | O | - | 13 | 6 |  | 9 | 6 | 2 |
| *Protaetia metallica* | Scarabaeidae | H | - | 2 | 0 |  | 0 | 0 | 2 |
| *Cyphon padi* | Scirtidae | H | - | 0 | 0 |  | 0 | 1 | 2 |
| *Cyphon variabilis* | Scirtidae | H | - | 6 | 8 |  | 1 | 0 | 2 |
| *Byrrhus fasciatus* | Byrrhidae | H | - | 2 | 1 |  | 1 | 0 | 2 |
| *Eanus costalis* | Elateridae | H | - | 327 | 328 |  | 165 | 295 | 18 |
| *Orithales serraticornis* | Elateridae | NA | - | 18 | 6 |  | 17 | 11 | 4, 24 |
| *Pheletes aeneoniger* | Elateridae | H | - | 1 | 1 |  | 0 | 0 | 2 |
| *Selatosomus melancholicus* | Elateridae | H | - | 0 | 1 |  | 3 | 4 | 2 |
| *Podabrus alpinus* | Cantharidae | P | - | 2 | 0 |  | 0 | 0 | 2 |
| *Rhagonycha elongata* | Cantharidae | P | - | 0 | 0 |  | 2 | 0 | 2, 10 |
| *Rhagonycha nigriventris* | Cantharidae | P | - | 1 | 0 |  | 0 | 0 | 2 |
| *Necrobia violacea* | Cleridae | O | - | 0 | 0 |  | 1 | 0 | 2 |
| *Phalacrus substriatus* | Phalacridae | F | - | 0 | 0 |  | 1 | 0 | 2 |
| *Atomaria apicalis* | Cryptophagidae | F | - | 2 | 5 |  | 0 | 0 | 2 |
| *Atomaria hislopi* | Cryptophagidae | F | - | 0 | 1 |  | 0 | 0 | 2, 14 |
| *Atomaria nitidula* | Cryptophagidae | F | - | 0 | 1 |  | 0 | 0 | 2 |
| *Atomaria peltataeformis* | Cryptophagidae | F | - | 0 | 0 |  | 0 | 1 | 20 |
| *Coccinella trifasciata* | Coccinellidae | P | - | 1 | 1 |  | 0 | 0 | 2 |
| *Nephus bipunctatus* | Coccinellidae | P | - | 0 | 0 |  | 0 | 4 | 2 |
| *Gonioctena intermedia* | Chrysomelidae | H | - | 0 | 3 |  | 0 | 0 | 2 |
| *Phratora vitellinae* | Chrysomelidae | H | - | 0 | 1 |  | 1 | 0 | 2 |
| *Coeliodinus rubicundus* | Curculionidae | H | - | 1 | 8 |  | 1 | 0 | 2 |
| *Polydrusus fulvicornis* | Curculionidae | H | - | 1 | 41 |  | 0 | 45 | 2, 21 |
| d) Unknown dead wood  association |  |  |  |  |  |  |  |  |  |
| *Acrotona amblystegii* | Staphylinidae | NA | - | 0 | 0 |  | 3 | 1 | NA |
| *Atheta altaica* | Staphylinidae | NA | - | 0 | 0 |  | 0 | 1 | NA |
| *Atheta brunneipennis* | Staphylinidae | P | - | 4 | 1 |  | 2 | 1 | 1, 2 |
| *Atheta celata* | Staphylinidae | P | - | 1 | 0 |  | 0 | 0 | 2, 4 |
| Unknown dead wood  association (continued) |  |  |  | Kirkenes | |  | Tana | |  |
| Species | Family | Guild | Birch | D | L |  | D | L | Ref. |
| *Atheta euryptera* | Staphylinidae | P | - | 0 | 0 |  | 2 | 0 | 1, 4 |
| *Atheta sodalis* | Staphylinidae | F | - | 1 | 0 |  | 0 | 0 | 1, 4 |
| *Atheta subtilis* | Staphylinidae | F | - | 3 | 4 |  | 3 | 10 | 2, 4 |
| *Bisnius puella* | Staphylinidae | P | - | 0 | 1 |  | 3 | 0 | 2, 4 |
| *Boreophilia islandica* | Staphylinidae | P | - | 0 | 0 |  | 5 | 2 | NA |
| *Encephalus complicans* | Staphylinidae | P | - | 0 | 1 |  | 0 | 0 | 1, 4 |
| *Liogluta alpestris* | Staphylinidae | P | - | 2 | 0 |  | 0 | 0 | 2 |
| *Mycetoporus erichsonanus* | Staphylinidae | F | - | 0 | 0 |  | 1 | 0 | 2, 22 |
| *Stenus hyperboreus* | Staphylinidae | P | - | 0 | 0 |  | 1 | 0 | NA |
| *Neohypdonus arcticus* | Elateridae | NA | - | 0 | 1 |  | 0 | 0 | NA |
| *Dichelotarsus lapponicus* | Cantharidae | P | - | 0 | 0 |  | 0 | 3 | 23 |
| *Corticarina minuta* | Latridiidae | F | - | 0 | 0 |  | 1 | 0 | 2, 4 |
| *Cortinicara gibbosa* | Latridiidae | F | - | 0 | 2 |  | 0 | 0 | 4, 13 |
| *Latridius porcatus* | Latridiidae | F | - | 0 | 1 |  | 9 | 1 | 2, 4 |

**References (Table S1)**

1. Dahlberg A, Stokland JN (2004) Vedlevande arters krav på substrat: sammanställning och analys av 3 600 arter. Skogsstyrelsen.

2. Böhme J (2005) Die Käfer Mitteleuropas: Katalog. Elsevier Spektrum Akademischer Verlag, München

3. Kålås JA, Viken Å, Henriksen S, Skjelseth S (2010) The 2010 Norwegian Red List for Species. Norwegian Biodiversity Information Centre, Norway

4. The Saproxylic Database ([www.saproxylic.org](http://www.saproxylic.org)), accessed on 14 February 2013

5. Gärdenfors U (2010) 2010 red list of Swedish species. ArtDatabanken-SLU i samarbete med Naturvårdsverket.

6. Gibb H, Pettersson RB, Hjältén J, Hilszczański J, Ball JP, Johansson T, Atlegrim O, Danell K (2006) Conservation-oriented forestry and early successional saproxylic beetles: Responses of functional groups to manipulated dead wood substrates. Biological Conservation 129 (4):437-450. doi:<http://dx.doi.org/10.1016/j.biocon.2005.11.010>

7. Johansson T, Olsson J, Hjältén J, Jonsson BG, Ericson L (2006) Beetle attraction to sporocarps and wood infected with mycelia of decay fungi in old-growth spruce forests of northern Sweden. Forest Ecology and Management 237 (1–3):335-341. doi:<http://dx.doi.org/10.1016/j.foreco.2006.09.056>

8. Langor DW, Spence JR, Hammond HJ (2004) Saproxylic beetles (Coleoptera) using Populus in boreal aspen stands of western Canada: spatiotemporal variation and conservation of assemblages. Canadian Journal of Forest Research 34 (1):1-19

9. Alexander KNA, Anderson R (2012) The Beetles of Decaying Wood in Ireland: A Provisional Annotated Checklist of Saproxylic Coleoptera. Ireland, National Parks and Wildlife Service

10. Hansen V, Larsson SG (1973) (2. edition) Danmarks fauna; illustrerede haandbøger over den danske dyreverden. Bd.44 (Biller, X. Blødvinger, Klannere M.M). G.E.C. Gad., København

11. Buckland P.I. & Buckland P.C. (2006). Bugs Coleopteran Ecology Package (Versions: BugsCEP v7.63; Bugsdata v8.0; BugsMCR v2.02; BugStats v1.22) [Downloaded 21 September 2013] www.bugscep.com.

12. Hjältén J, Stenbacka F, Pettersson RB, Gibb H, Johansson T, Danell K, Ball JP, Hilszczański J (2012) Micro and Macro-Habitat Associations in Saproxylic Beetles: Implications for Biodiversity Management. Plos One 7 (7):e41100. doi:10.1371/journal.pone.0041100

13. Olsson J, Johansson T, Jonsson BG, Hjältén J, Edman M, Ericson L (2012) Landscape and substrate properties affect species richness and community composition of saproxylic beetles. Forest Ecology and Management 286 (0):108-120. doi:<http://dx.doi.org/10.1016/j.foreco.2012.08.033>

14. Hyvärinen E (2006) Green-tree retention and controlled burning in restoration and conservation of beetle diversity in boreal forests. Ph. D thesis, Faculty of Forestry, University of Joensuu

15. Lindroth CH (1992) Ground beetles (Carabidae) of Fennoscandia : a zoogeographic study. Part. 1: Specific knowledge regarding the species Smithsonian Institution Libraries and National Science Foundation, Washington, D.C. doi:10.5962/bhl.title.46300

16. Sundt E (1958) Revision of the Fenno-Scandian species of the genus Acrotrichis. Norsk Entomologisk tidsskrift 10 (4-5):241-278

17. Hansen V (1968) (2. edition) Danmarks fauna; illustrerede haandbøger over den danske dyreverden. Bd.77 (Biller, XXV. Ådselbiller, Stumpbiller, M.M). G.E.C. Gad., København

18. Johansson T, Andersson J, Hjalten J, Dynesius M, Ecke F (2011) Short-term responses of beetle assemblages to wildfire in a region with more than 100 years of fire suppression. Insect Conservation and Diversity 4 (2):142-151

19. Cuccodoro G, Löbl I (1997) Revision of the Palaearctic rove beetles of the genus Megarthrus Curtis (Coleoptera: Staphylinidae: Proteininae). Journal of natural history 31 (9):1347-1415

20. Huggert L, Ulefors SO (1971) Anteckningar om svenska Coleoptera. Entomologisk tidskrift 92 (1-2): 54-65

21. Hansen V, Larsson SG (1965) Danmarks fauna; illustrerede haandbøger over den danske dyreverden. Bd.69 (Biller, XXI. Snudebiller). G.E.C. Gad., København

22. Landin BO (1970) Fältfauna. Insekter 2, del 1 och 2. (Coleoptera, Strepsiptera, Hymenoptera). Natur och Kultur, Stockholm

23. Fender KM (1973) Ecological Notes on Podabrus (Coleoptera: Cantharidae). The Coleopterists Bulletin 27 (1):11-17. doi:10.2307/3999623

24. Gibb H, Johansson T, Stenbacka F, Hjältén J (2013) Functional Roles Affect Diversity-Succession Relationships for Boreal Beetles. Plos One 8 (8):e72764. doi:10.1371/journal.pone.0072764

**Table S2**. Results from model selection based on QAIC_c_ for log-linear models relating total counts of obligate- facultative- and non-saproxylic species to the predictors forest damage, location and year. The selected models are highlighted in bold. K = number of parameters. ∆_i_ = Difference in QAIC_c_ between model i and the model with the lowest QAIC_c_. *w*_i_ = QAIC_c_ weight. D = damage. L = Location. Y = Year. Y×D = Year × Damage interaction. L×D = Location × Damage interaction. L×Y = Location × Year interaction. Note that the estimate of the overdispersion parameter counts as an additional parameter in the models.

| Model | | | | | | |  | Obligate saproxylic | | |  | Facultative saproxylic | | |  | Non-saproxylic | | |
| --- | --- | --- | --- | --- | --- | --- | --- | --- | --- | --- | --- | --- | --- | --- | --- | --- | --- | --- |
| D | L | Y | Y×D | L×D | L×Y | K |  | QAIC_c_ | ∆_i_ | *w*_i_ |  | QAIC_c_ | ∆_i_ | *w*_i_ |  | QAIC_c_ | ∆_i_ | *w*_i_ |
| × | × | × | × | × | × | 8 |  | 76.04 | 5.14 | 0.04 |  | 90.42 | 1.35 | 0.21 |  | 69.59 | 0.85 | 0.27 |
| × | × | × | × | × |  | 7 |  | 73.85 | 2.95 | 0.13 |  | 90.66 | 1.59 | 0.25 |  | 71.54 | 2.80 | 0.10 |
| × | × | × | × |  | × | 7 |  | 80.54 | 9.64 | 0 |  | 103.78 | 14.70 | 0 |  | 87.47 | 18.73 | 0 |
| × | × | × |  | × | × | 7 |  | 72.89 | 1.99 | 0.20 |  | **89.08** | **0** | **0.55** |  | **68.74** | **0** | **0.41** |
| × | × | × | × |  |  | 6 |  | 78.60 | 7.70 | 0.01 |  | 107.16 | 18.08 | 0 |  | 88.05 | 19.31 | 0 |
| × | × | × |  | × |  | 6 |  | **70.90** | **0** | **0.55** |  | 90.66 | 1.59 | 0.25 |  | 70.10 | 1.36 | 0.21 |
| × | × | × |  |  | × | 6 |  | 77.64 | 6.74 | 0.02 |  | 105.57 | 16.50 | 0 |  | 85.25 | 16.51 | 0 |
| × | × | × |  |  |  | 5 |  | 75.82 | 4.92 | 0.05 |  | 107.33 | 18.26 | 0 |  | 86.78 | 18.04 | 0 |
| × | × |  |  | × |  | 5 |  | 92.88 | 21.98 | 0 |  | 114.09 | 25.01 | 0 |  | 77.82 | 9.08 | 0 |
| × | × |  |  |  |  | 4 |  | 97.97 | 27.07 | 0 |  | 130.91 | 41.84 | 0 |  | 94.66 | 25.92 | 0 |
| × |  | × | × |  |  | 5 |  | 100.68 | 29.78 | 0 |  | 104.49 | 15.41 | 0 |  | 104.11 | 35.37 | 0 |
| × |  | × |  |  |  | 4 |  | 98.06 | 27.16 | 0 |  | 104.82 | 15.74 | 0 |  | 103.00 | 34.25 | 0 |
| × |  |  |  |  |  | 3 |  | 120.35 | 49.45 | 0 |  | 128.55 | 39.47 | 0 |  | 111.02 | 42.28 | 0 |

**Table S3**. Coefficients from selected log-linear and logistic models relating total counts and proportions, respectively, of obligate- facultative- and non-saproxylic species to the predictors forest damage, location and year. The intercept in all models represents living forest in the Kirkenes transect in 2011. 95 % confidence intervals are given in brackets and statistically significant terms are highlighted in bold. Significance codes: *** = p ≤ 0.001; ** = p ≤ 0.01; * = p ≤ 0.05; . = p ≤ 0.1.

| Log-linear models |  | Obligate saproxylic | Facultative saproxylic | Non-saproxylic |
| --- | --- | --- | --- | --- |
| Intercept |  | 2.65 [2.19, 3.10] | 2.04 [1.53, 2.55] | 4.19 [3.84, 4.54] |
| Damage (Dead) |  | **1.23 [0.78, 1.68]** *** | 0.42 [-0.11, 0.95] | **0.76 [0.39, 1.14]** *** |
| Location (Tana) |  | -0.13 [-0.74, 0.48] | 0.08 [-0.74, 0.90] | -0.33 [-0.95, 0.29] |
| Year (2012) |  | **0.88 [0.51, 1.25]** *** | **0.59 [0.04, 1.14]** * | 0.25 [-0.12, 0.62] |
| Location (Tana) × Year (2012) |  | - | 0.88 [0.01, 1.76] **.** | 0.65 [0.00, 1.31] **.** |
| Location (Tana) × Damage (Dead) |  | **-1.08 [-1.83, -0.32]** ** | **-1.63 [-2.41, -0.85]** *** | **-1.33 [-1.95, -0.71]** *** |
| Logistic models |  | Obligate saproxylic | Facultative saproxylic | Non-saproxylic |
| Intercept |  | -1.52 [-1.87, -1.17] | -2.76 [-3.31, -2.22] | 0.67 [0.32, 1.02] |
| Damage (Dead) |  | **0.63 [0.19, 1.06] **** | 0.03 [-0.69, 0.74] | -0.18 [-0.53, 0.16] |
| Location (Tana) |  | - | **0.39 [0.01, 0.78] *** | - |
| Year (2012) |  | - | **0.81 [0.21, 1.41] *** | -0.36 [-0.72, 0.00] **.** |
| Year (2012) × Damage (Dead) |  | - | **-0.96 [-1.81, -0.10] *** | - |

**Table S4**. Results from model selection based on QAIC_c_ for logistic models relating total counts of obligate- facultative- and non-saproxylic species to the predictors forest damage, location and year. The selected models are highlighted in bold. K = number of parameters. ∆_i_ = Difference in QAIC_c_ between model i and the model with the lowest QAIC_c_. *w*_i_ = QAIC_c_ weight. D = damage. L = Location. Y = Year. Y×D = Year × Damage interaction. L×D = Location × Damage interaction. L×Y = Location × Year interaction. Note that the estimate of the overdispersion parameter counts as an additional parameter in the models.

| Model | | | | | | |  | Obligate saproxylic | | |  | Facultative saproxylic | | |  | Non-saproxylic | | |
| --- | --- | --- | --- | --- | --- | --- | --- | --- | --- | --- | --- | --- | --- | --- | --- | --- | --- | --- |
| D | L | Y | Y×D | L×D | L×Y | K |  | QAIC_c_ | ∆_i_ | *w*_i_ |  | QAIC_c_ | ∆_i_ | *w*_i_ |  | QAIC_c_ | ∆_i_ | *w*_i_ |
| × | × | × | × | × | × | 8 |  | 71.28 | 5.62 | 0.01 |  | 89.10 | 5.04 | 0.02 |  | 75.34 | 4.28 | 0.02 |
| × | × | × | × | × |  | 7 |  | 72.33 | 6.67 | 0.01 |  | 86.69 | 2.63 | 0.06 |  | 76.37 | 5.30 | 0.01 |
| × | × | × | × |  | × | 7 |  | 68.82 | 3.16 | 0.05 |  | 86.18 | 2.11 | 0.08 |  | 72.20 | 1.13 | 0.11 |
| × | × | × |  | × | × | 7 |  | 68.54 | 2.87 | 0.05 |  | 90.01 | 5.95 | 0.01 |  | 74.29 | 3.22 | 0.04 |
| × | × | × | × |  |  | 6 |  | 70.11 | 4.45 | 0.02 |  | **84.06** | **0** | **0.22** |  | 73.41 | 2.35 | 0.06 |
| × | × | × |  | × |  | 6 |  | 69.69 | 4.03 | 0.03 |  | 88.33 | 4.27 | 0.03 |  | 74.93 | 3.87 | 0.03 |
| × | × | × |  |  | × | 6 |  | 66.27 | 0.61 | 0.16 |  | 87.68 | 3.62 | 0.04 |  | 71.33 | 0.27 | 0.17 |
| × | × | × |  |  |  | 5 |  | 67.55 | 1.89 | 0.09 |  | 86.10 | 2.04 | 0.08 |  | 72.16 | 1.10 | 0.11 |
| × | × |  |  | × |  | 5 |  | 69.37 | 3.71 | 0.03 |  | 88.58 | 4.51 | 0.02 |  | 76.06 | 4.99 | 0.02 |
| × | × |  |  |  |  | 4 |  | 67.26 | 1.59 | 0.10 |  | 86.67 | 2.61 | 0.06 |  | 73.48 | 2.42 | 0.06 |
| × |  | × | × |  |  | 5 |  | 68.52 | 2.86 | 0.05 |  | 85.19 | 1.13 | 0.12 |  | 71.97 | 0.91 | 0.12 |
| × |  | × |  |  |  | 4 |  | 66.06 | 0.39 | 0.18 |  | 87.90 | 3.84 | 0.03 |  | **71.07** | **0** | **0.19** |
| × |  |  |  |  |  | 3 |  | **65.66** | **0** | **0.22** |  | 89.52 | 5.46 | 0.01 |  | 73.02 | 1.95 | 0.07 |

**Table S5**. Results from model selection based on QAIC_c_ for log-linear models relating counts of individual beetle species to the predictors forest damage, location and year. The selected models are highlighted in bold. K = number of parameters. ∆_i_ = Difference in QAIC_c_ between model i and the model with the lowest QAIC_c_. *w*_i_ = QAIC_c_ weight. D = Damage. L = Location. Y = Year. Y×D = Year × Damage interaction. L×D = Location × Damage interaction. L×Y = Location × Year interaction. Note that the estimate of the overdispersion parameter counts as an additional parameter in the models.

| Model | | | | | | |  | *Elateroides dermestoides* | | |  | *Rabocerus foveolatus* | | |  | *Podistra schoenherri* | | |
| --- | --- | --- | --- | --- | --- | --- | --- | --- | --- | --- | --- | --- | --- | --- | --- | --- | --- | --- |
| D | L | Y | Y×D | L×D | L×Y | K |  | QAIC_c_ | ∆_i_ | *w*_i_ |  | QAIC_c_ | ∆_i_ | *w*_i_ |  | QAIC_c_ | ∆_i_ | *w*_i_ |
| × | × | × | × | × | × | 8 |  | 65.64 | 5.85 | 0.02 |  | 99.30 | 5.60 | 0.02 |  | 95.97 | 7.64 | 0.01 |
| × | × | × | × | × |  | 7 |  | 62.51 | 2.73 | 0.11 |  | 96.17 | 2.47 | 0.10 |  | 92.88 | 4.55 | 0.03 |
| × | × | × | × |  | × | 7 |  | 66.51 | 6.73 | 0.01 |  | 97.54 | 3.84 | 0.05 |  | 95.33 | 7.00 | 0.01 |
| × | × | × |  | × | × | 7 |  | 62.68 | 2.90 | 0.10 |  | 97.75 | 4.05 | 0.04 |  | 93.84 | 5.52 | 0.02 |
| × | × | × | × |  |  | 6 |  | 63.64 | 3.85 | 0.06 |  | 94.67 | 0.96 | 0.20 |  | 92.59 | 4.26 | 0.03 |
| × | × | × |  | × |  | 6 |  | **59.78** | **0** | **0.42** |  | 95.03 | 1.33 | 0.17 |  | 90.96 | 2.63 | 0.07 |
| × | × | × |  |  | × | 6 |  | 63.81 | 4.03 | 0.06 |  | 96.24 | 2.54 | 0.09 |  | 93.56 | 5.23 | 0.02 |
| × | × | × |  |  |  | 5 |  | 61.08 | 1.30 | 0.22 |  | **93.70** | **0** | **0.33** |  | 90.84 | 2.52 | 0.07 |
| × | × |  |  | × |  | 5 |  | 77.96 | 18.17 | 0 |  | 105.05 | 11.35 | 0 |  | **88.33** | **0** | **0.26** |
| × | × |  |  |  |  | 4 |  | 79.42 | 19.63 | 0 |  | 103.88 | 10.18 | 0 |  | 88.37 | 0.05 | 0.26 |
| × |  | × | × |  |  | 5 |  | 84.60 | 24.81 | 0 |  | 110.19 | 16.49 | 0 |  | 93.28 | 4.95 | 0.02 |
| × |  | × |  |  |  | 4 |  | 82.20 | 22.42 | 0 |  | 109.39 | 15.68 | 0 |  | 91.69 | 3.36 | 0.05 |
| × |  |  |  |  |  | 3 |  | 100.68 | 40.90 | 0 |  | 119.71 | 26.01 | 0 |  | 89.36 | 1.04 | 0.16 |

Table S5 continued.

| Model | | | | | | |  | *Malthodes guttifer* | | |  | *Liotrichus affinis* | | |  | *Oxytelus laqueatus* | | |
| --- | --- | --- | --- | --- | --- | --- | --- | --- | --- | --- | --- | --- | --- | --- | --- | --- | --- | --- |
| D | L | Y | Y×D | L×D | L×Y | K |  | QAIC_c_ | ∆_i_ | *w*_i_ |  | QAIC_c_ | ∆_i_ | *w*_i_ |  | QAIC_c_ | ∆_i_ | *w*_i_ |
| × | × | × | × | × | × | 8 |  | 70.15 | 7.21 | 0.02 |  | 77.07 | 1.45 | 0.23 |  | 78.26 | 7.66 | 0.01 |
| × | × | × | × | × |  | 7 |  | 67.01 | 4.06 | 0.08 |  | 77.77 | 2.16 | 0.16 |  | 75.98 | 5.38 | 0.03 |
| × | × | × | × |  | × | 7 |  | 84.29 | 21.34 | 0 |  | 88.96 | 13.34 | 0 |  | 75.13 | 4.53 | 0.04 |
| × | × | × |  | × | × | 7 |  | 67.32 | 4.38 | 0.06 |  | **75.61** | **0** | **0.48** |  | 75.35 | 4.76 | 0.04 |
| × | × | × | × |  |  | 6 |  | 81.62 | 18.68 | 0 |  | 94.05 | 18.43 | 0 |  | 73.03 | 2.43 | 0.12 |
| × | × | × |  | × |  | 6 |  | 64.52 | 1.58 | 0.26 |  | 78.47 | 2.86 | 0.12 |  | 73.37 | 2.78 | 0.10 |
| × | × | × |  |  | × | 6 |  | 81.94 | 18.99 | 0 |  | 91.89 | 16.27 | 0 |  | 72.40 | 1.81 | 0.17 |
| × | × | × |  |  |  | 5 |  | 79.31 | 16.36 | 0 |  | 94.92 | 19.31 | 0 |  | **70.60** | **0** | **0.41** |
| × | × |  |  | × |  | 5 |  | **62.94** | **0** | **0.58** |  | 103.52 | 27.91 | 0 |  | 103.49 | 32.90 | 0 |
| × | × |  |  |  |  | 4 |  | 77.89 | 14.95 | 0 |  | 120.13 | 44.51 | 0 |  | 100.88 | 30.28 | 0 |
| × |  | × | × |  |  | 5 |  | 78.87 | 15.93 | 0 |  | 91.62 | 16.01 | 0 |  | 76.86 | 6.27 | 0.02 |
| × |  | × |  |  |  | 4 |  | 76.72 | 13.77 | 0 |  | 92.65 | 17.04 | 0 |  | 74.59 | 3.99 | 0.06 |
| × |  |  |  |  |  | 3 |  | 75.44 | 12.50 | 0 |  | 118.01 | 42.39 | 0 |  | 105.01 | 34.41 | 0 |

Table S5 continued.

| Model | | | | | | |  | *Anthophagus* *omalinus* | | |  | *Eanus costalis* | | |  | *Polydrusus fulvicornis* | | |
| --- | --- | --- | --- | --- | --- | --- | --- | --- | --- | --- | --- | --- | --- | --- | --- | --- | --- | --- |
| D | L | Y | Y×D | L×D | L×Y | K |  | QAIC_c_ | ∆_i_ | *w*_i_ |  | QAIC_c_ | ∆_i_ | *w*_i_ |  | QAIC_c_ | ∆_i_ | *w*_i_ |
| × | × | × | × | × | × | 8 |  | 60.67 | 2.83 | 0.09 |  | 85.70 | 3.43 | 0.09 |  | 74.17 | 4.58 | 0.02 |
| × | × | × | × | × |  | 7 |  | 58.07 | 0.23 | 0.32 |  | 84.90 | 2.62 | 0.13 |  | 76.52 | 6.92 | 0.01 |
| × | × | × | × |  | × | 7 |  | 69.85 | 12.01 | 0 |  | 102.50 | 20.22 | 0 |  | 72.28 | 2.68 | 0.06 |
| × | × | × |  | × | × | 7 |  | 60.74 | 2.90 | 0.09 |  | 83.18 | 0.90 | 0.31 |  | 71.68 | 2.08 | 0.08 |
| × | × | × | × |  |  | 6 |  | 66.90 | 9.06 | 0 |  | 101.36 | 19.08 | 0 |  | 74.43 | 4.84 | 0.02 |
| × | × | × |  | × |  | 6 |  | **57.84** | **0** | **0.36** |  | **82.28** | **0** | **0.48** |  | 73.91 | 4.32 | 0.03 |
| × | × | × |  |  | × | 6 |  | 69.56 | 11.72 | 0 |  | 99.64 | 17.37 | 0 |  | **69.59** | **0** | **0.22** |
| × | × | × |  |  |  | 5 |  | 66.84 | 9.00 | 0 |  | 98.92 | 16.64 | 0 |  | 72.00 | 2.41 | 0.07 |
| × | × |  |  | × |  | 5 |  | 59.90 | 2.06 | 0.13 |  | 156.29 | 74.01 | 0 |  | 72.61 | 3.02 | 0.05 |
| × | × |  |  |  |  | 4 |  | 69.06 | 11.22 | 0 |  | 173.08 | 90.81 | 0 |  | 70.86 | 1.26 | 0.12 |
| × |  | × | × |  |  | 5 |  | 84.23 | 26.39 | 0 |  | 102.67 | 20.39 | 0 |  | 73.31 | 3.71 | 0.04 |
| × |  | × |  |  |  | 4 |  | 84.33 | 26.49 | 0 |  | 100.39 | 18.11 | 0 |  | 71.04 | 1.44 | 0.11 |
| × |  |  |  |  |  | 3 |  | 86.70 | 28.86 | 0 |  | 174.70 | 92.42 | 0 |  | 70.04 | 0.44 | 0.18 |

**Table S6**. Coefficients from selected log-linear models relating counts of individual beetle species to the predictors forest damage, location and year. The intercept in all models represents living forest in the Kirkenes transect in 2011. 95 % confidence intervals are given in brackets and statistically significant terms are highlighted in bold. Significance codes: *** = p ≤ 0.001; ** = p ≤ 0.01; * = p ≤ 0.05; . = p ≤ 0.1.

| Species |  | *Elateroides dermestoides* | *Rabocerus foveolatus* | *Podistra schoenherri* |
| --- | --- | --- | --- | --- |
| DWA group/Trophic guild |  | Obligate sx./Fungivore | Obligate sx./Predator | Obligate sx./Predator |
| Intercept |  | -0.20 [-1.67, 1.27] | 0.64 [0.04, 1.24] | 0.81 [0.20, 1.42] |
| Damage (Dead) |  | **3.23** [1.83, 4.62] *** | **0.99** [0.44, 1.53] *** | 0.20 [-0.70, 1.11] |
| Location (Tana) |  | 0.48 [-1.40, 2.36] | -**1.07** [-1.62, -0.51] *** | **0.94** [0.17, 1.70] * |
| Year (2012) |  | **1.45** [0.74, 2.17] *** | **0.88** [0.34, 1.43] ** | - |
| Location (Tana) × Year (2012) |  | - | - | - |
| Location (Tana) × Damage (Dead) |  | -**2.15** [-4.16, -0.14] * | - | -0.97 [-2.13, 0.19] |
| Species |  | *Malthodes guttifer* | *Liotrichus affinis* | *Oxytelus laqueatus* |
| DWA group/Trophic guild |  | Obligate sx.^1^/Predator | Facultative sx./? | Non-sx./Predator |
| Intercept |  | 0.77 [-0.13, 1.68] | 1.57 [0.88, 2.27] | -1.25 [-2.38, -0.13] |
| Damage (Dead) |  | **1.19** [0.10, 2.28] * | 0.65 [-0.02, 1.33] **.** | **2.02** [1.18, 2.87] *** |
| Location (Tana) |  | **1.29** [0.21, 2.37] * | -0.14 [-1.45, 1.16] | -**0.75** [-1.34, -0.17] * |
| Year (2012) |  | - | **0.73** [0.02, 1.44] * | **2.06** [1.17, 2.96] *** |
| Location (Tana) × Year (2012) |  | - | **1.40** [0.13, 2.67] * | - |
| Location (Tana) × Damage (Dead) |  | -**3.34** [-5.21, -1.48] *** | -**2.04** [-3.03, -1.05] *** | - |
| Species |  | *Anthophagus omalinus* | *Eanus costalis* | *Polydrusus fulvicornis* |
| DWA group/Trophic guild |  | Non-sx./Predator | Non-sx./Herbivore | Non-sx./Herbivore |
| Intercept |  | 3.78 [3.26, 4.31] | 2.27 [1.83, 2.71] | 1.42 [0.67, 2.17] |
| Damage (Dead) |  | **0.88** [0.26, 1.51] ** | 0.40 [0.00, 0.81] **.** | -**4.55** [-8.32, -0.77] * |
| Location (Tana) |  | -0.18 [-1.00, 0.63] | 0.30 [-0.12, 0.71] | -0.62 [-2.08, 0.83] |
| Year (2012) |  | -**0.58** [-1.13, -0.03] * | **1.53** [1.13, 1.94] *** | -0.39 [-1.57, 0.79] |
| Location (Tana) × Year (2012) |  | - | - | 1.77 [-0.06, 3.60] **.** |
| Location (Tana) × Damage (Dead) |  | -**2.15** [-3.51, -0.80] ** | -**1.39** [-2.03, -0.74] *** | - |

^1^ Not associated with birch.


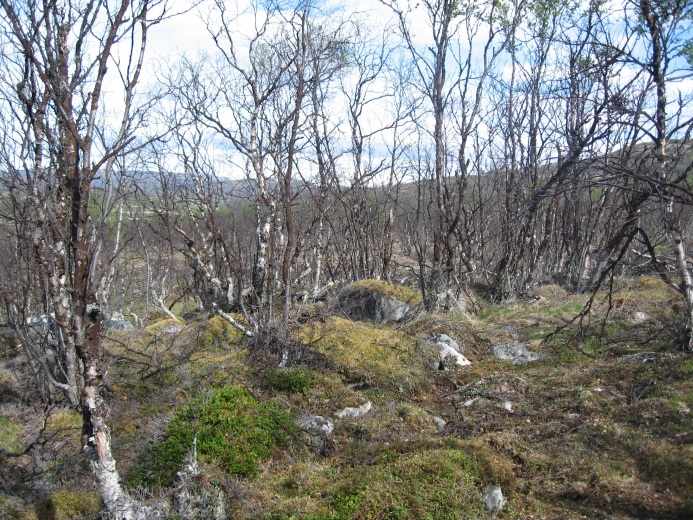

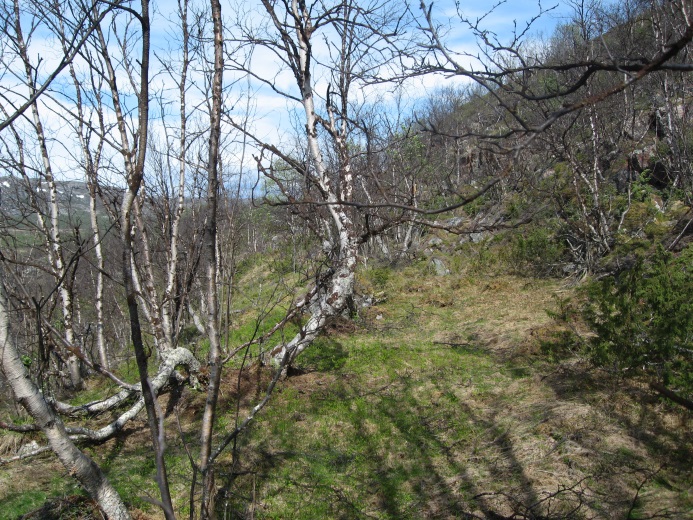

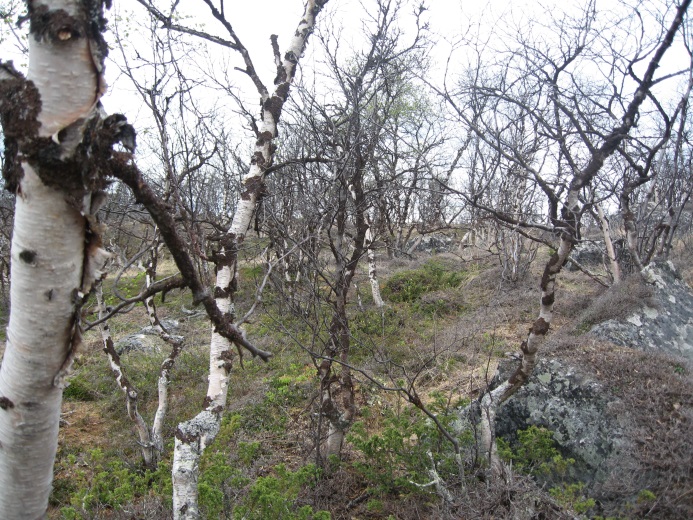

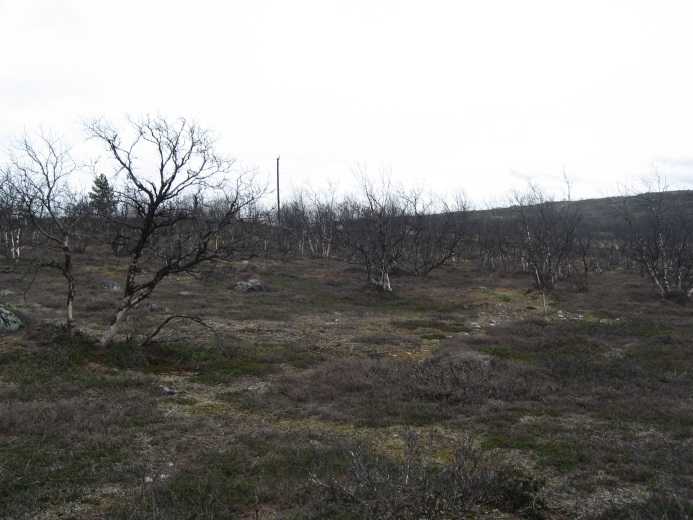

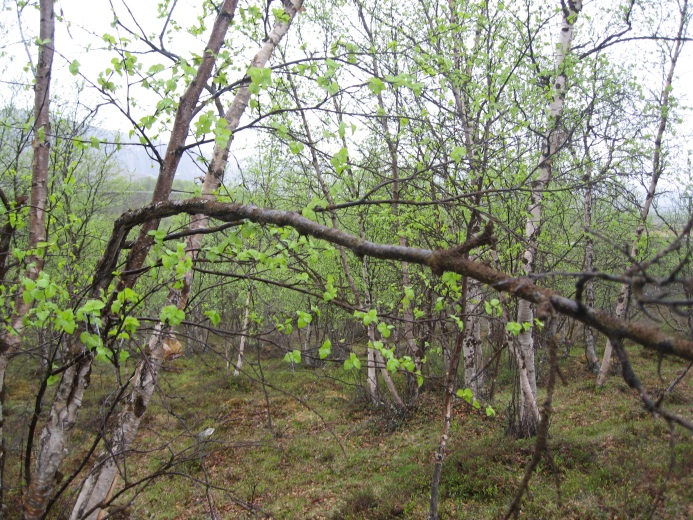

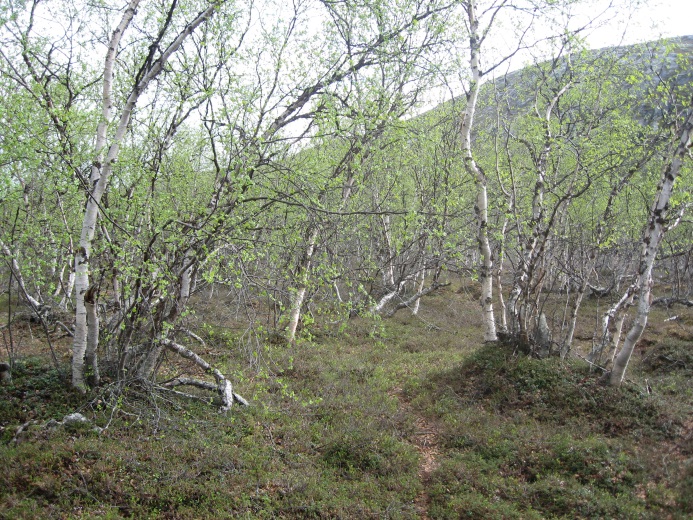

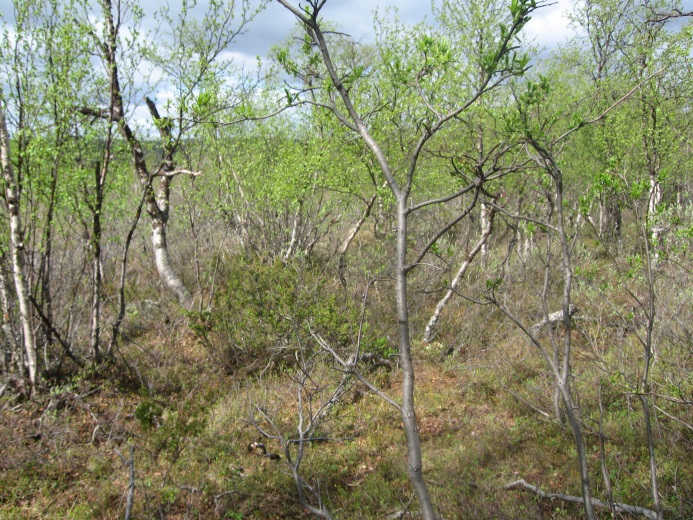

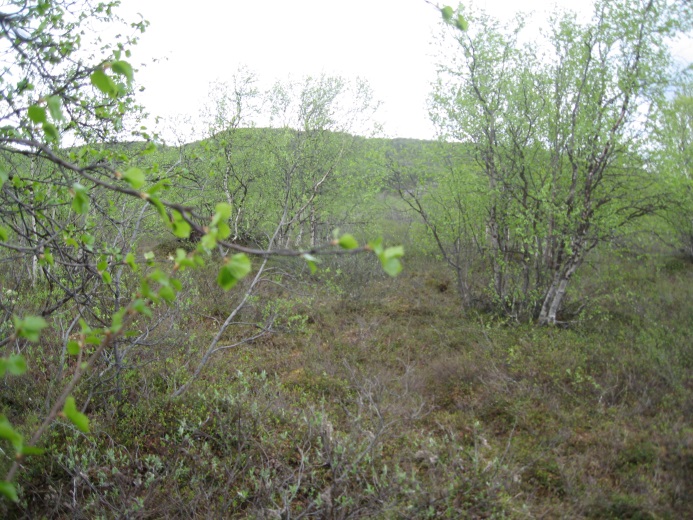

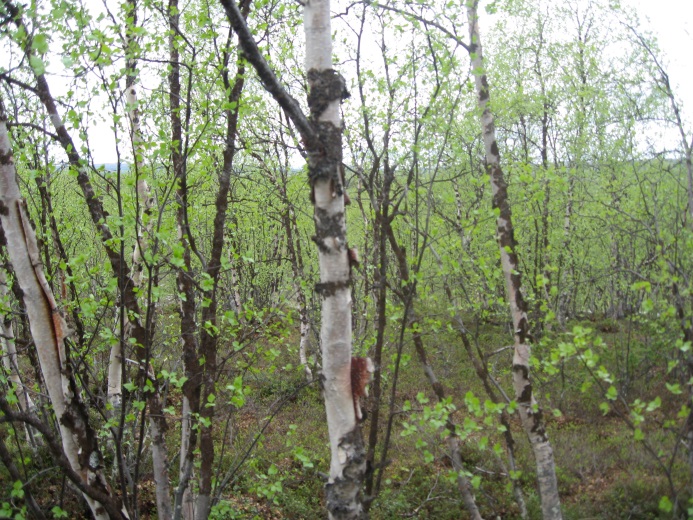

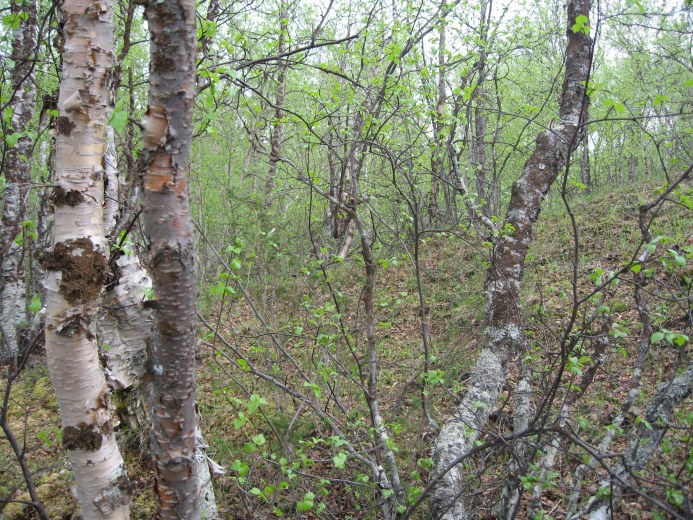


**Ki 1**

**Ki 2**

**Ki 3**

**Ki 4**

**Ki 5**

**Ki 6**

**Ki 7**

**Ki 8**

**Ki 9**

**Ki 10**


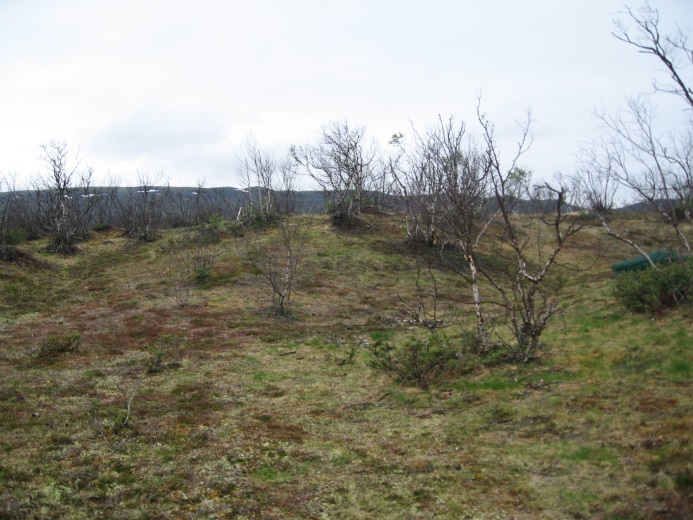

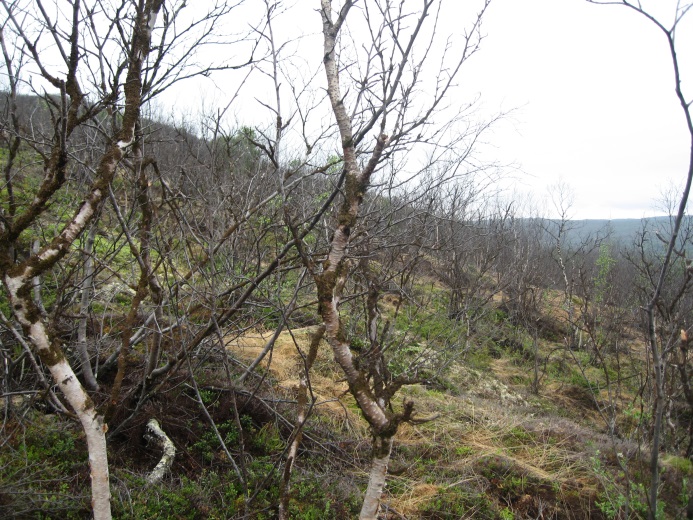

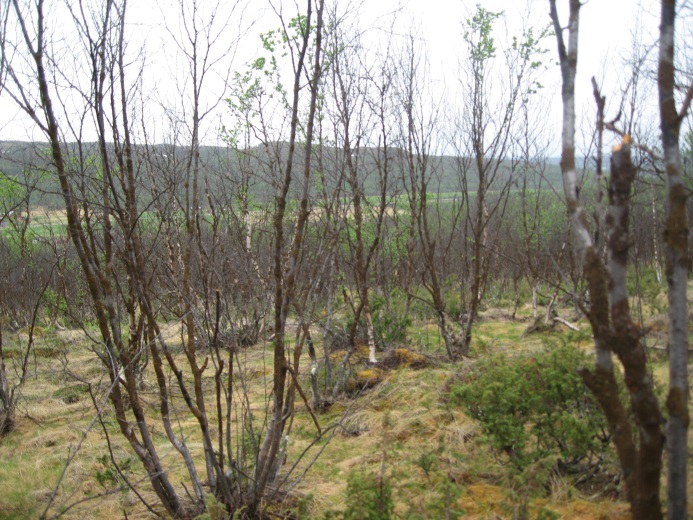

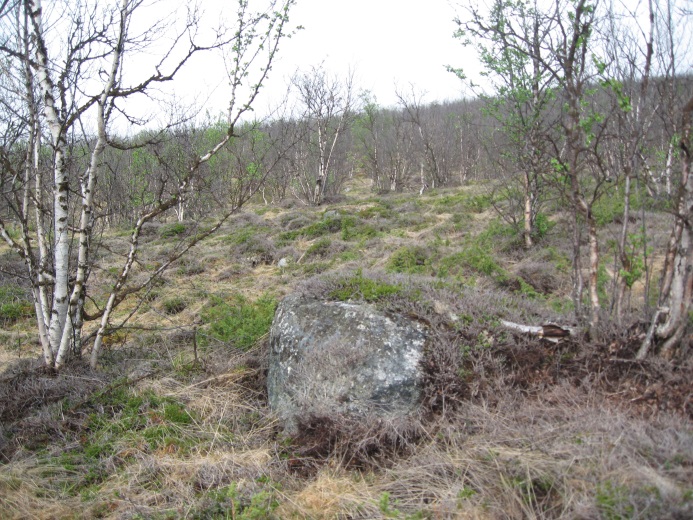

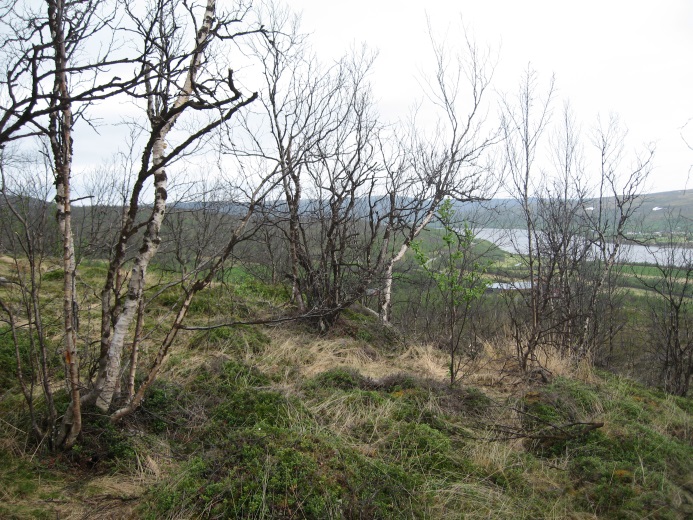

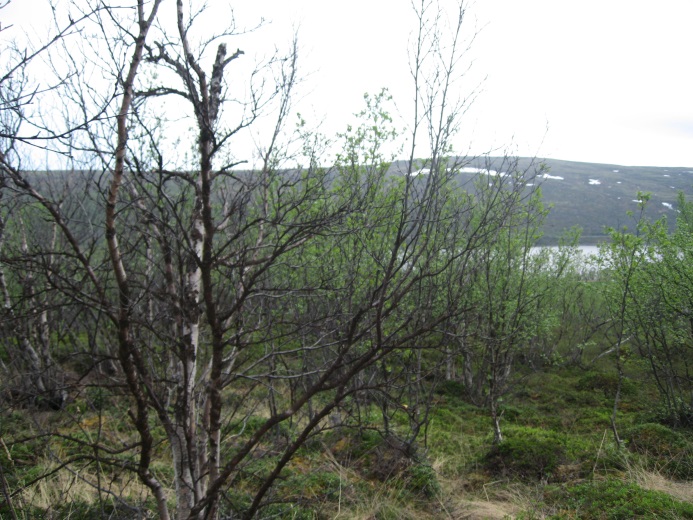

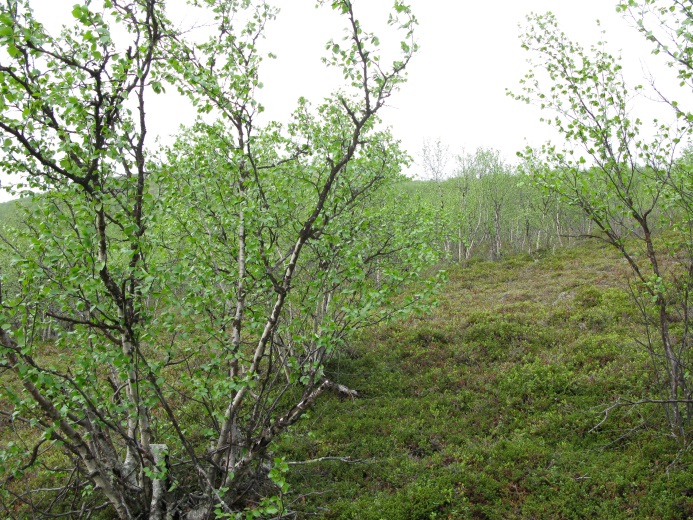

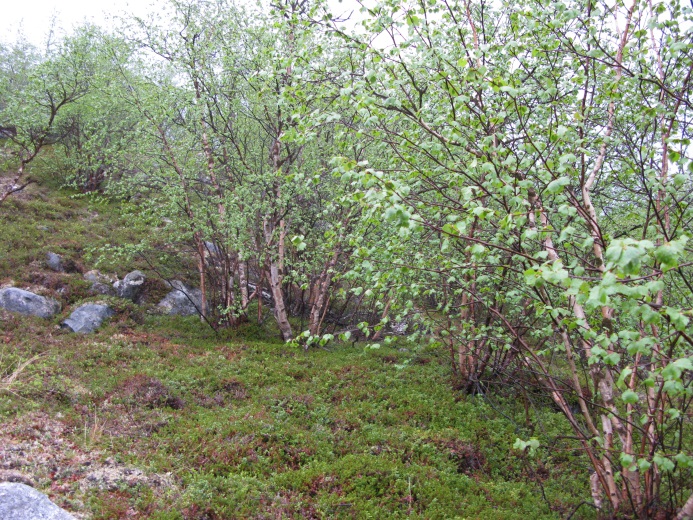

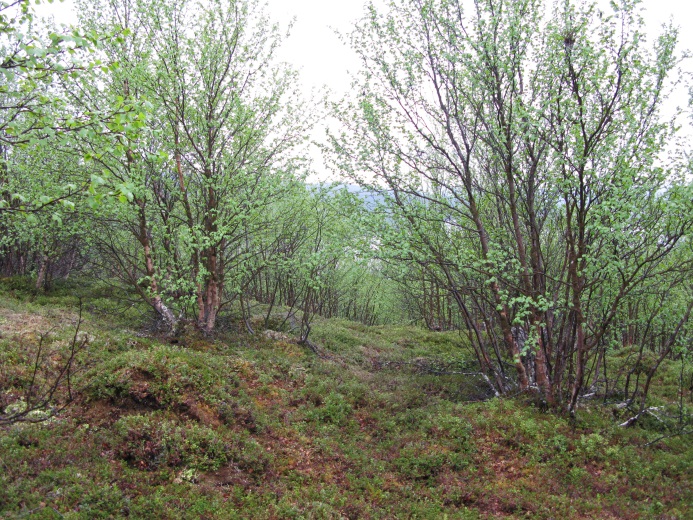

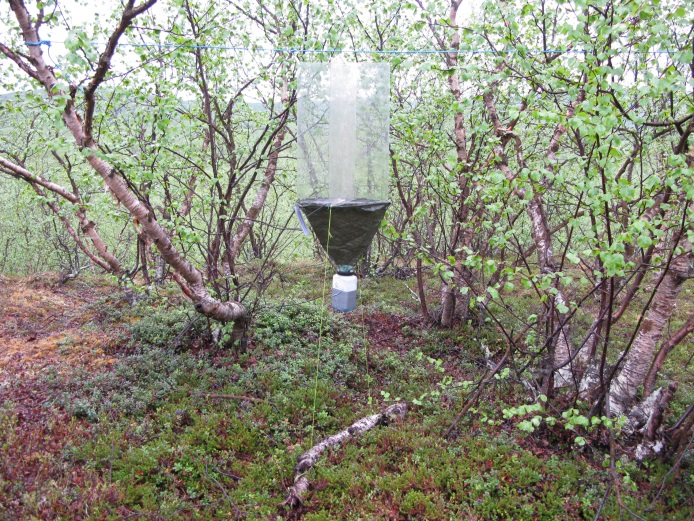


**Ta 1**

**Ta 2**

**Ta 3**

**Ta 4**

**Ta 5**

**Ta 6**

**Ta 7**

**Ta 8**

**Ta 9**

**Ta 10**

**Figure S1**. Photographs from the individual sampling stations in Kirkenes (Ki) and Tana (Ta) in early June 2011, just prior to mounting of the window traps. The photograph from station 10 in Tana also shows a mounted trap in a typical position.

**Ta 9**

**Ta 10**


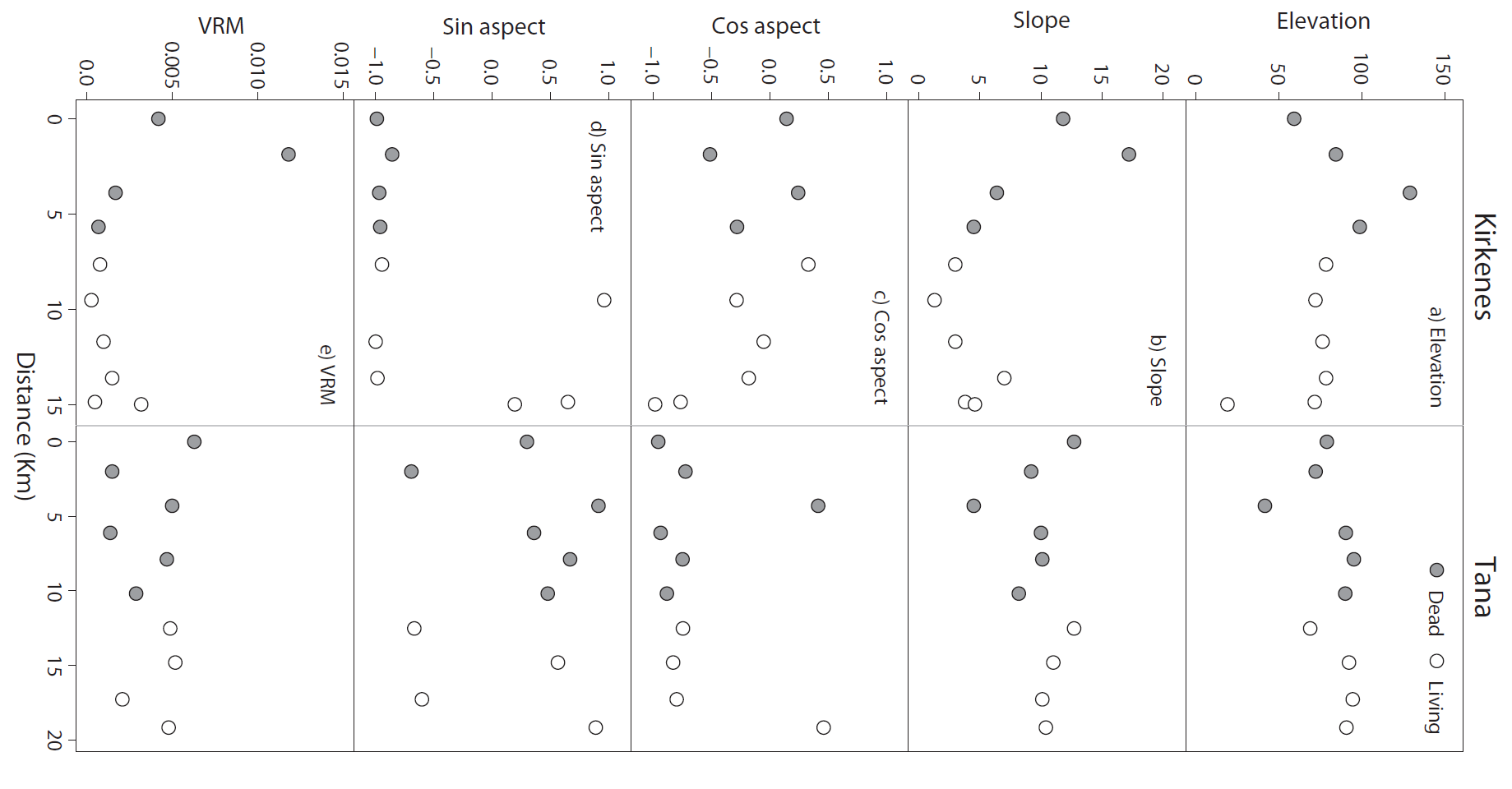


**Figure S2**. Station-specific scores for topographic variables plotted against the airline distance from the first station in the Kirkenes and Tana transects. The scores were extracted from a digital elevation model with 20 m pixel size and represent means for a 200 m neighbourhood around each station. a) Elevation. b) Slope. c) Cosine aspect, describing the “northness” of slopes (1 = facing north, -1 = facing south). d) Sine aspect, describing the “eastness” of slopes (1 = facing east, -1 = facing west). e) Vector ruggedness measure (VRM). A measure that incorporates heterogeneity in both slope and aspect [1]. Grey points = Stations with dead forest. White pointes = Stations with living forest.

**References (Figure S2)**

1. Sappington JM, Longshore KM, Thompson DB (2007) Quantifying Landscape Ruggedness for Animal Habitat Analysis: A Case Study Using Bighorn Sheep in the Mojave Desert. The Journal of Wildlife Management 71 (5):1419-1426. doi:10.2307/4496214
